# Genome-wide mapping of the *Galleria mellonella* transcription start sites during fungal infection and treatment

**DOI:** 10.1016/j.isci.2026.117675

**Published:** 2026-10-01

**Authors:** Imad Abugessaisa, Mickey Konings, Ri-ichiroh Manabe, Michihira Tagami, Jessica Severin, Akira Hasegawa, Tsugumi Kawashima, Hiroko Kinoshita, Shohei Noma, Chitose Takahashi, Annelies Verbon, Yasushi Okazaki, Wendy W.J. van de Sande, Takeya Kasukawa

**Affiliations:** 1 Premium Research Institute for Human Metaverse Medicine (WPI-PRIMe), The University of Osaka, 2-2, Yamadaoka, Suita, Osaka 565-0871, Japan; 2 Graduate School of Medicine, The University of Osaka, 2-2, Yamadaoka, Suita, Osaka 565-0871, Japan; 3 Laboratory for Large-Scale Biomedical Data Technology, RIKEN Center for Integrative Medical Sciences, 1-7-22 Suehiro-cho, Tsurumi-ku, Yokohama City, Kanagawa 230-0045, Japan; 4 Erasmus MC, University Medical Center Rotterdam, Department of Medical Microbiology and Infectious Diseases, Dr. Molewaterplein 40, 3015GD Rotterdam, the Netherlands; 5 Laboratory for Comprehensive Genomic Analysis, RIKEN Center for Integrative Medical Sciences, 1-7-22 Suehiro-cho, Tsurumi-ku, Yokohama City, Kanagawa 230-0045, Japan; 6 UMC Utrecht, Department of Internal Medicine, Heidelberglaan 100, Utrecht, the Netherlands

**Keywords:** galleria mellonella, transcription start site, promoters, enhancers, cap analysis of gene expression

## Abstract

*Galleria mellonella* is an emerging experimental model, but its current genome annotation lacks transcription start site (TSS) information. Because TSS annotation is essential for understanding transcriptional regulation, we used low quantity single strand CAGE (LQ-ssCAGE) to map cap analysis of gene expression (CAGE)-defined TSSs from 72 *G. mellonella* larval samples. Among these, 36 larvae were infected with the fungal pathogen *Madurella mycetomatis* and 36 served as healthy controls, allowing identification of TSSs under both infected and healthy conditions. Infected and healthy larvae were treated with itraconazole or ravuconazole. To assess key characteristics of the CAGE data, TSS peaks and putative enhancers were intersected with the existing genome annotation for annotation and characterization. This analysis identified two sets of TSS peaks, permissive and robust, representing the first draft TSS atlas for *G. mellonella*. The atlas-derived annotations showed a markedly stronger core promoter sequence signature than the NCBI reference annotation, supporting improved resolution and accuracy of TSS annotation in the atlas.

## Highlights

• Performed CAGE profiling on healthy, infected, and treated *G. mellonella* larvae
• Identified transcription start site (TSS) peaks and putative enhancers
• Identified TSSs displayed characteristic features of high-quality CAGE data
• Integrated TSS annotation into the existing NCBI *G. mellonella* genome annotation

## Graphical abstract

**Figure:**
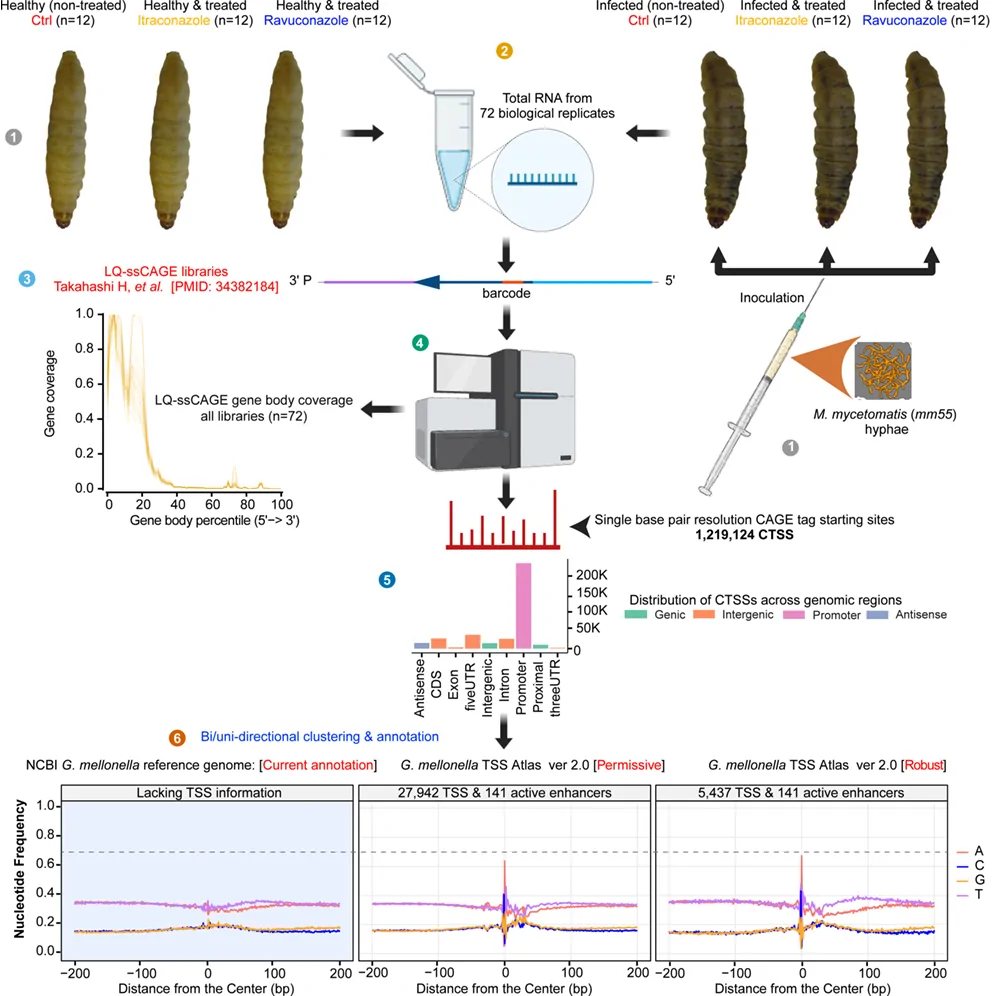

## Teaser

Genomics; Biocomputational method; Computational bioinformatics

## Introduction

The greater wax moth *Galleria mellonella* (Linnaeus, 1758) is an invertebrate that belongs to the Pyralidae family. Recently, *G. mellonella* has gained increasing attention as disease model in biological research (Figure S1). The larvae have been used extensively in *in vivo* infection studies, including disease modeling, the investigation of host-pathogen interactions,^1^ the characterization of innate immune responses, and the assessment of novel drug efficacy through susceptibility testing.^2^^,^^3^ As an infection model, the model enabled the study of infectious disease phenotypes that are difficult to study and characterize in human patients.^4^ Using *G. mellonella* as a disease model in biological research has several benefits. First, it is cheap and easy to host, has a short life cycle with many offspring, and does not require ethical approval. As a research model, *G. mellonella* reduces the use of vertebrates in biomedical research, which is much more costly.

The first genome of *G. mellonella* was sequenced in 2017 and contained 1,937 contigs (genome assembly ASM258982v1), and this was followed by several initiatives to enhance the genome assembly and provided gene annotation utilizing the latest development in long-read sequencing. The current NCBI reference genome assembly of *G. mellonella* (CSIRO_AGI_GalMel_v1) has well improved and has 99.3% lepidoptera_odb10 BUSCO (Figure S2) and includes 15,138 genes and pseudogenes (Figure S3).^5^ The availability of a high-quality genome assembly will enable studies to understand the structure and function of the genome model.^6^ Genome annotation can either be obtained from public databases or via computational analysis and prediction.^6^

Together with the availability of reference genomes, gene and feature annotations are provided by RefSeq,^7^ UCSC Genome Browser,^8^ and NCBI.^9^ Novel biological insights, such as understanding transcriptional regulation and RNA production, can be obtained by utilizing these resources. The instructions for gene regulation determine when, where, and at which level a gene or set of genes transcribed in a specific cell; these instructions are encoded in *cis*-regulatory elements (CREs). Promoters, enhancers, and silencers are CREs and contain transcription factor (TF) binding sites (TFBS). The gene expression levels in cell types are determined by the TFs together with different transcriptional regulators.^10^ In general, CREs can be divided into two categories: constitutively open CREs and CREs accessible in a cell-type-specific manner.^11^ The transcription start site (TSS) is the specific genomic region in which transcription is initiated. TSSs are dual regulated by TFs and promoters.^12^ The genomic region of the TSS in the genome makes it the most suitable unit to integrate various types of data related to transcriptional events.^12^ Mapping and characterization of TSS is required to understand transcription regulation and to understand how TFs, chromatin proteins, and TFBSs interact with promoter regions to regulate transcription.^13^

Methods for mapping TSSs began with primer extension studies^14^ followed by rapid amplification of cDNA ends, for example, RACE, which is a technique used to clone full-length transcript and transcript variants,^15^^,^^16^ as well as the expressed sequence tag (EST) approach for cap-trapped 5′ end EST sequencing.^17^ Cap analysis of gene expression (CAGE)^18^ and its subsequent developments^19^^,^^20^^,^^21^^,^^22^ precisely map the 5′ end cap of the transcript. In addition to the TSS peaks, CAGE data allow for the prediction of active enhancers. Other methods include Start-seq,^23^ csRNA-seq,^24^ and GRO^25^/PRO-cap.^26^ A comprehensive review of the assays to map TSS promoter and active enhancers is provided in Yao et al.^27^ and Core et al.^28^ With the proceeding information in mind, the main analysis of CAGE data involves the clustering of CAGE tags; such analysis considers identifying a group of CAGE transcription start site (CTSS) clusters corresponding to the expression activity of a single TSS and related enhancers.^29^

To perform genome-wide mapping and annotation of TSSs in *G. mellonella* we used RNA isolated from *G. mellonella* larvae then prepared and sequenced two types of sequencing libraries, namely, RNA-seq and LQ-ssCAGE (*n* = 72). RNA-seq from similar samples was used to validate the quality of the LQ-ssCAGE mapping. LQ-ssCAGE identifies the TSSs of the 5′ end capped RNA, and at the same time measures the expression level of the transcript.^21^ *G. mellonella* is an effective model for analyzing disease burden and response to treatment; we infected *G. mellonella* larvae with the fungal pathogen *Madurella mycetomatis* (NCBI:txid100816). *M. mycetomatis* is a high-priority fungal pathogen^30^ and belongs to the order of the Sordariales in the Sordariomycetes family and is the most common causative agent of eumycetoma. Eumycetoma is an neglected tropical disease (NTD) characterized by tumorous lesions in the subcutaneous tissue.^31^ To cure eumycetoma, patients are usually treated with a combination of antifungal treatment and surgery.^32^ Currently, the antifungal agent itraconazole is the drug of choice, along with ravuconazole, which was a drug recently assessed in the first clinical trial for eumycetoma.^33^ Therefore, to mimic a natural infection and treatment, we divided the *M. mycetomatis-*infected larvae into three groups. One control group was treated with solvent only, one group was treated with itraconazole, and one group with ravuconazole. LQ-ssCAGE (*n* = 72) and RNA-seq (*n* = 72) libraries were deeply sequenced using next-generation sequencing platforms, which resulted in a large 5′ end, promoter-rich dataset for *G. mellonella*. The LQ-ssCAGE data enable us to identify the CTSS; TSSs, characterized by uni-directional clusters; and enhancers, characterized by bi-directional clusters. We analyzed the TSS shapes, and confirmed the known quality of promoters, including the GC content and TATA box upstream the promoter region. We identified interacting TSSs and enhancers as TSSs and enhancers that are both in proximity to each other and highly correlate in their expression.^34^ We consider the top 5% highly expressed TSS peaks and performed *de novo* motif analysis.

We provide a genomic view of *G. mellonella* new annotation via the ZENBU genome browser.^35^ This enables the user to browse the genomic location of the TSS peaks visually. Also, users will be able to upload any gene expression data generated from *G. mellonella* to the ZENBU genome browser moving forward. CAGE TSS, tag cluster (TC), TSS peaks (permissive and robust), and putative enhancers are provided via Mendeley data and the raw sequencing data provided via NCBI GEO database.

Considering the lack of available TSS information for *G. mellonella* and availability of technology to map TSS, it motivated us to map TSS information and identified high-confidence TSSs, constituting the first draft TSS atlas for *G. mellonella*. The atlas will contribute to the gene annotation of *G. mellonella* and encourage the continued usage of the model in biomedical research.

## Results

### Generation and primary processing of RNA-seq and CAGE tag 5′ end

Total RNA was extracted from non-infected and infected larvae after 6, 30, 72, and 168 h incubation at 37°C (Figure 1A). Total RNA passed QC (260/280 and 260/230) (Table S1) used for the preparation of two types of sequencing libraries, RNA-seq and LQ-ssCAGE. For the paired-end (PE) LQ-ssCAGE, Read1 was the cap-trapped 5′ reads from the transcript start site and Read2 was the sample-barcode sequence and random priming site read. Using only Read1, we retained a total of ∼53.3 million reads. We counted the total sequence tags from the LQ-ssCAGE fastq across all samples, the majority of sequence libraries contained about 5 × 10^5^ to 1 × 10^6^ sequence tags. Then, we tested their density distribution across all samples. The density plot demonstrated a tightly clustered profile (indicates no occurrence of outlier or major batch effects). The unimodal distribution of the density plot indicates consistent sequencing depth across samples (Figure 1B). Raw sequence data from RNA-seq and LQ-ssCAGE were processed using Moirai workflow^36^ (Figure S4) and tested for gene body coverage.^36^ We obtained a typical uniform gene body coverage pattern of RNA-seq and a typical strong 5′ end enrichment coverage pattern for LQ-ssCAGE (Figure 1C) (data and code availability). RNA-seq sequence reads and the cap-trapped 5′ tags were then mapped to the *G. mellonella* genome assembly CSIRO_AGI_GalMel_v1 and showed a high mapping rate after untemplated 5′ guanine (G) bases (LQ-ssCAGE only). We obtained comparable sequencing depth across conditions with minor variation between samples (Figure 1D) (data and code availability). RNA-seq libraries (*n* = 72) were used to confirm the quality of the LQ-ssCAGE dataset. We performed a pairwise correlation analysis between the normalized read counts (CPM) of the mapped RNA-seq reads and the normalized read counts (tags per million [TPM]) of the mapped cap-trapped 5′ end tags. Figure 1E shows a positive pairwise Spearman correlation between RNA-seq and corresponding LQ-ssCAGE samples indicating good separation between assay type but not between biological conditions.

**Figure 1:**
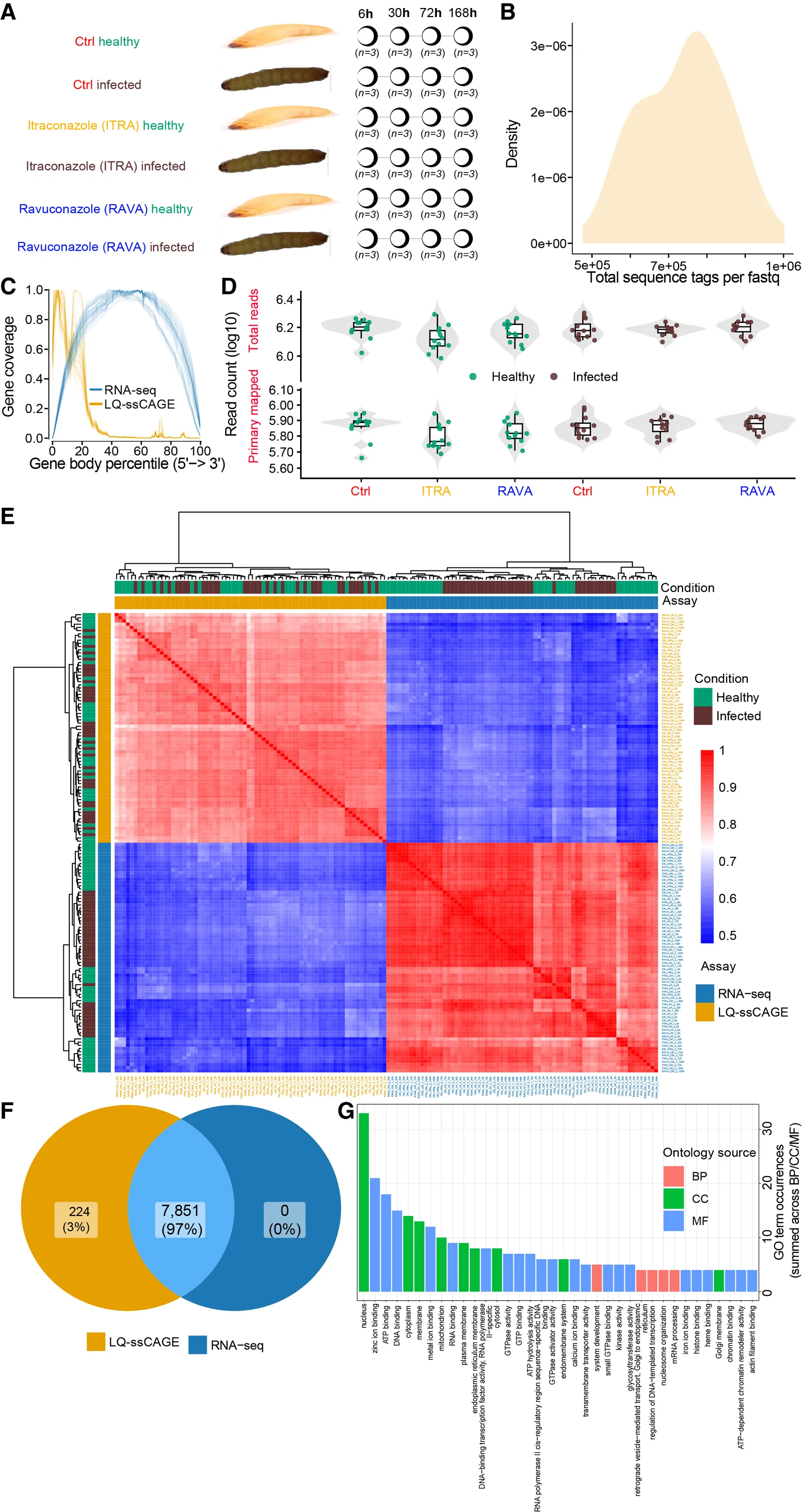
*G*. *mellonella* larvae sample preparation and transcriptomics quality (A) Study design, larvae infection, and treatment. A total of 72 *Galleria mellonella* (NCBI:txid7137) larvae samples were processed. Half of the samples (*n* = 36) were infected with the pathogen *Madurella mycetomatis* (NCBI:txid100816; strain *mm55*), while the remaining samples (*n* = 36) used as uninfected controls (Ctrl). Three experimental sets were prepared: (1) untreated controls (Ctrl healthy and Ctrl infected), (2) itraconazole-treated larvae (itraconazole healthy and itraconazole infected), and (3) ravuconazole-treated larvae (ravuconazole healthy and ravuconazole infected). For each condition, three biological replicates were collected at four time points (6, 30, 72, and 168 h). Total RNA was extracted and used to prepare both LQ-ssCAGE (*n* = 72) and RNA-seq (*n* = 72) libraries. (B) Density distribution of total LQ-ssCAGE sequence tags calculated from raw sequence files. It shows a clear central tendency total 53,356,962 sequence tags obtained from all libraries. (C) Gene body coverage of the mapped sequence reads for LQ-ssCAGE and RNA-seq libraries computed and visualized by SkewC untemplated 5′ guanine (G) bases were removed from LQ-ssCAGE reads. Total of 53,356,962 sequence tags were aligned to the *G. mellonella* reference genome assembly CSIRO_AGI_GalMel_v1. Prior to mapping, untemplated 5′ G bases were removed from LQ-ssCAGE reads. Each line represents one library. LQ-ssCAGE libraries show enrichment at the 5′ end of transcripts, whereas RNA-seq libraries display uniform coverage across gene bodies with a peak near the transcript center. (D) Distribution of mapped LQ-ssCAGE reads (total reads and primary mapped reads) shown as violin plots. Each violin represents a treatment condition or untreated control. Samples are grouped into healthy and infected categories. Boxplots indicate the median and interquartile range, and points represent individual libraries, colored by infection status. (E) Pairwise correlation heatmap of normalized read counts from RNA-seq (log2 CPM) and LQ-ssCAGE (log2 TPM) libraries. Samples are clustered by assay type (RNA-seq or LQ-ssCAGE) and condition (healthy or infected). A strong positive Spearman correlation was observed. (F) Discordance analyses between LQ-ssCAGE and RNA-seq assays. The Venn diagram shows the number of detected genes per each assay. (G) Annotation of GO terms of the set of genes detected by LQ-ssCAGE. The annotation terms in three categories are BP, MF, and CC.

### Discordance analysis between LQ-ssCAGE and RNA-seq assays

Discordance analysis of genes detected by LQ-ssCAGE and RNA-seq (Figure 1F) identified 7,851 *G. mellonella* genes detected by both assays and 224 genes detected exclusively by LQ-ssCAGE. To gain insight into the functional properties of the LQ-ssCAGE-only genes, we retrieved their associated Gene Ontology (GO) annotations from UniProt and summarized the overall terms by GO ID (Figure 1G). The most prominent cellular component (CC) terms included nucleus, cytoplasm, membrane systems, and mitochondrion, with nucleus being the most frequent category. Molecular function (MF) and biological process (BP) terms were dominated by nucleotide and metal binding activities, including ATP binding, kinase activity, GTPase-related functions, and zinc/iron/calcium ion binding, together with regulatory functions linked to DNA binding, RNA polymerase II-associated transcription factor activity, chromatin binding, histone binding, ATP-dependent chromatin remodeling, RNA binding, mRNA processing, nucleosome organization, and regulation of DNA-templated transcription. Together, these results indicate that genes uniquely detected by LQ-ssCAGE are predominantly associated with gene-expression regulatory functions, spanning chromatin organization, transcriptional control, and RNA processing.

Together, these findings suggest that LQ-ssCAGE preferentially captures genes linked to regulatory layers of gene expression, including chromatin, transcriptional, and RNA-processing pathways.

### TSS and enhancer atlas for *G. mellonella*

To generate a TSS and enhancer atlas for the model organism *G. mellonella*, we established an analysis workflow comprising four major stages (Figure 2). First, primary processing of the LQ-ssCAGE dataset (*n* = 72), includes trimming of untemplated G, mapping raw sequence reads to the reference genome, and extraction and summarization of the 5′ end of the mapped reads. This step produced a set of strand-specific BigWig files. Second, basic clustering of 5′ end signals was used to quantify CTSS usage across all 72 samples, yielding 1,219,124 raw CTSSs (data and code availability). These were filtered to retain 332,601 biologically relevant CTSSs (data and code availability). Raw counts were normalized within each sample using TPM, and pooled TPM values across samples were used to identify and quantify unidirectional clusters, referred to as TCs. Third, filtered CTSSs were used to identify both unidirectional clusters (TCs) and bi-directional clusters representing candidate enhancers. In total, 40,200 TCs (data and code availability) were used to define TSS clusters, from which we generated two TSS peak sets based on expression support: a permissive set of 27,942 TSS peaks (data and code availability), requiring tag counts >0 in at least four samples, and a robust set of 5,437 TSS peaks, requiring tag counts >0 in all samples (data and code availability). Bi-directional clusters (*n* = 728) (data and code availability) were further filtered to retain non-exonic candidates located in intergenic or intronic regions, resulting in a final set of 141 active enhancers (data and code availability). Thus, users of the atlas can select either the permissive or robust annotation set depending on their analytical needs. Finally, to facilitate integration of the identified TSSs into downstream analyses, we generated updated gene transfer format (GTF) annotations by integrating the current NCBI gene annotation for the CSIRO_AGI_GalMel_v1 assembly with the permissive and robust TSS peak sets (data and code availability).

**Figure 2:**
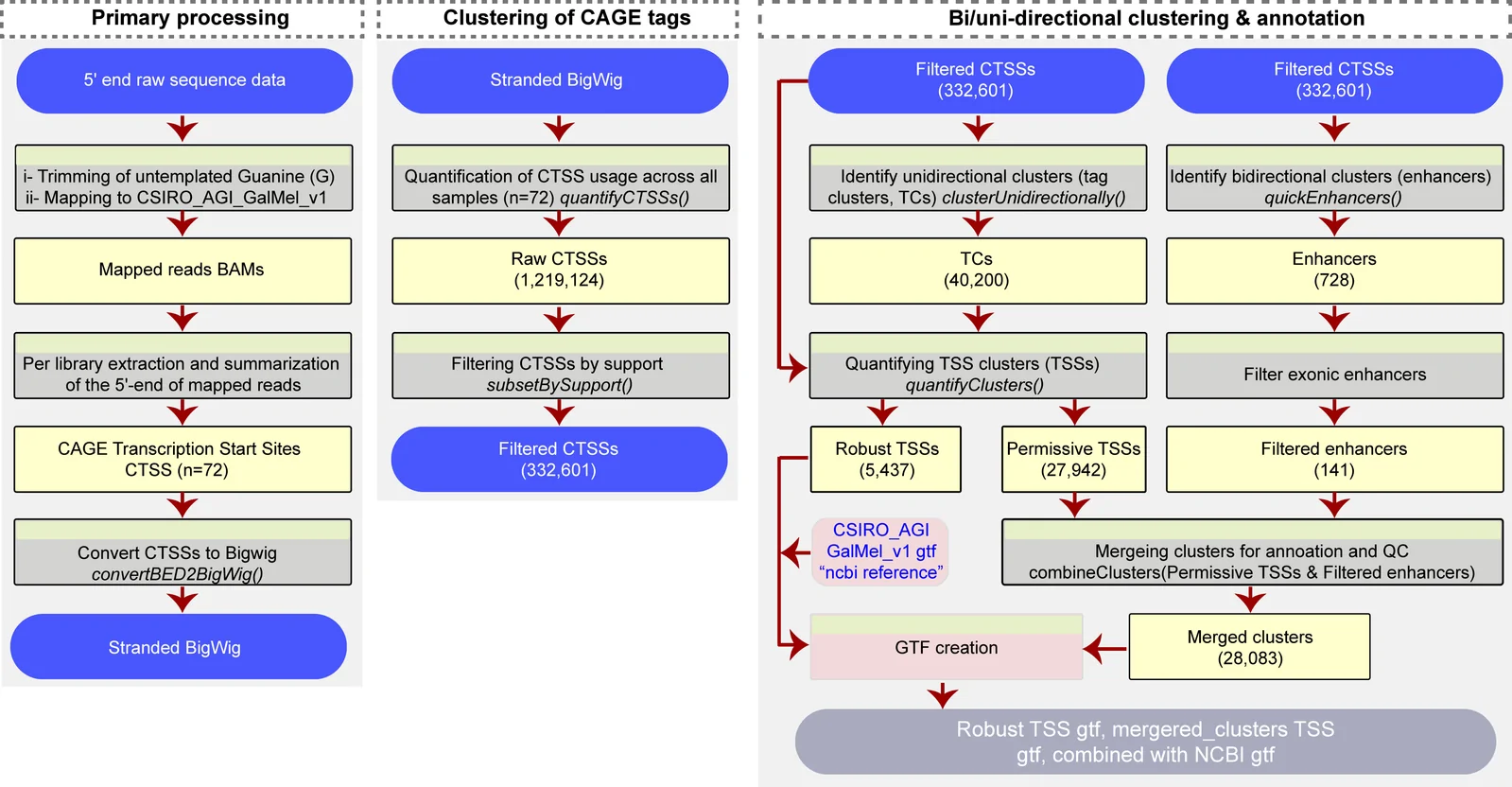
Schematic overview of the analysis workflow The analysis comprised four main stages: (1) primary processing of the 5′-end dataset (LQ-ssCAGE); (2) basic clustering to quantify CAGE transcription start site (CTSS) usage across all 72 samples; (3) identification of TSSs and active enhancers through uni- and bi-directional clustering; and (4) annotation of the resulting TSSs and active enhancers, followed by generation of the corresponding GTF files.

To assess the quality of the raw TCs, we first extracted the genomic coordinates of 5′ UTRs, CDS, introns, 3′ UTRs, and intergenic regions from the CSIRO_AGI_GalMel_v1 GTF annotation and intersected these features with the identified TCs. TCs were then ranked by pooled tag count. A large fraction of high-ranking TCs mapped to 5′ UTR-associated regions, whereas intergenic and intronic features tended to have lower tag counts. CDS and 3′ UTR features were found at the lowest ranks, indicating only a minor contribution to the overall tag signal (Figure 3A). We next examined overlaps between CAGE peaks, CAGE tags, and annotated gene features in *G. mellonella*. Among all CAGE peaks, 32.1% overlapped 5′ UTRs (Figure 3B), whereas 80.27% of all identified CAGE tags overlapped 5′ UTRs (Figure 3C). To further evaluate the association between TCs and annotated genes, we determined how many genes were linked to single versus multiple promoter-associated TCs, consistent with previous CAGE studies in *D. melanogaster*.^37^ We found that 5,278 genes (71.1%) were associated with multiple TCs, whereas 2,148 genes (28.9%) were associated with a single TC. Together, these results indicate that the identified TCs are strongly enriched at annotated promoter regions and capture the widespread use of alternative promoters in *G. mellonella*.

**Figure 3:**
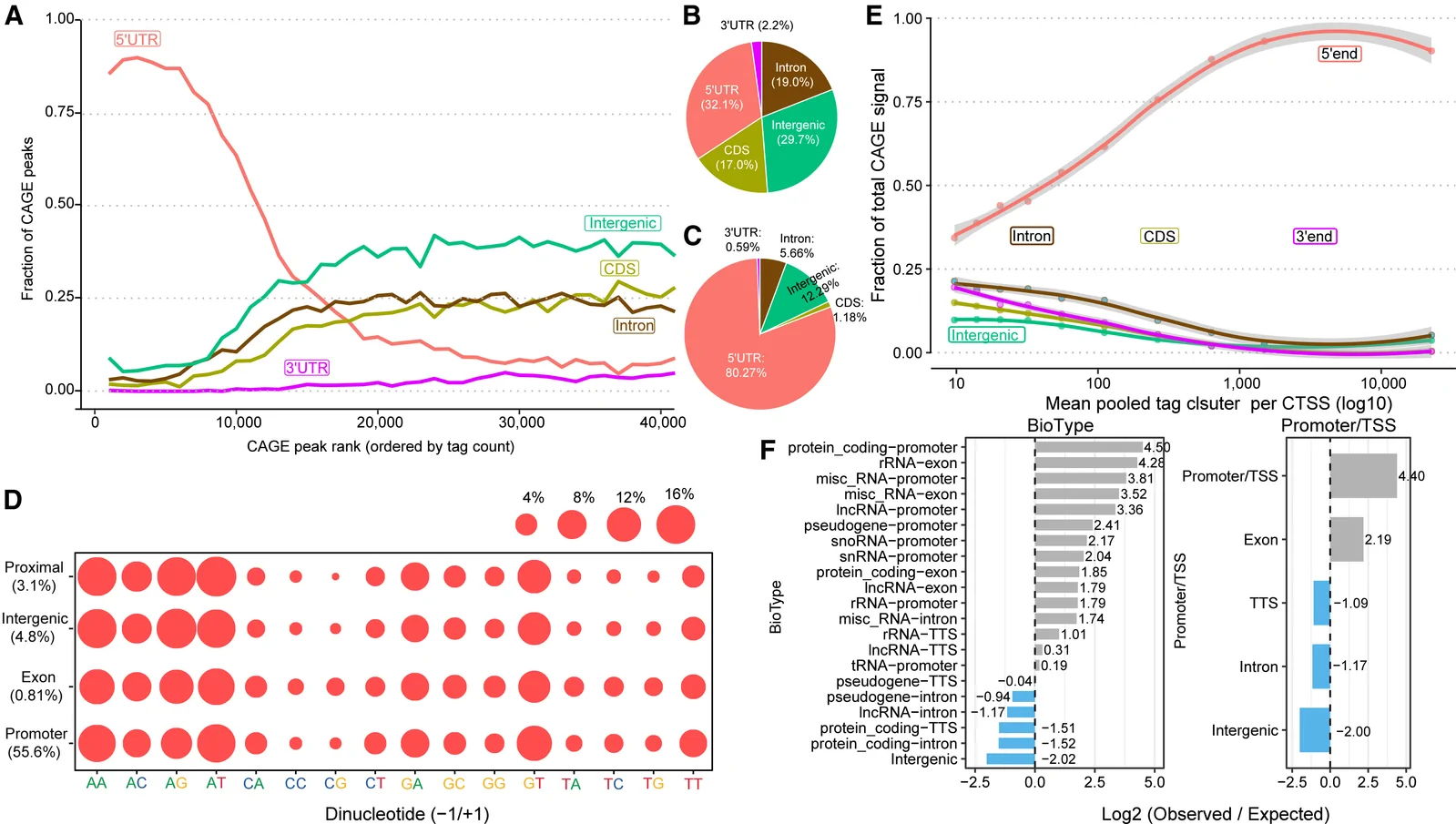
QC and characteristics of pooled CAGE TSSs (A) Fraction of CAGE peaks (CPs) overlapping genomic features per 1,000 peaks. To confirm the quality of the CPs, they are intersected against the *G. mellonella* latest gene model (CSIRO_AGI_GalMel_v1). The tag counts per CP are ordered in descending order. CPs are grouped into bins of 1,000, following the method used in (1), and the set of CPs with overlapping annotated features (five classes) is shown in the figure. The line plot indicates that CPs toward the top of the list overlap the 5′ UTR; in contrast, intergenic regions are at the bottom of the list of ranked CPs. (B) Fraction of CPs per genomic feature. (C) Fraction of total CAGE tags per feature. (D) Dinucleotide frequency analysis of dominant CTSSs. Dinucleotide composition for the dominant CTSS at the gene 5′ end promoter exhibits strong −1/+1 dinucleotide bias. (E) Genomic origin of CPs as a function of expression strength. Based on pooled CTSS expression (sum of TPM across all samples), CTSS were divided into 10 groups (dots overlay the line), for each group the fraction of total CAGE expression originating from five different genomic feature categories is shown as a function of the mean pooled expression per CTSS (log_10_). Smoothed trends with confidence intervals show enrichment of 5′ end-associated expressions at higher expression levels. Intergenic and 3′ end ranked at the bottom. (F) Concordance between defined TSS and the genome assembly CSIRO_AGI_GalMel_v1 gene annotation. To test the robustness of the defined TSS, HOMER was used to annotate the defined TSS peaks against CSIRO_AGI_GalMel_v1 gene annotation (GTF). The distance-based histogram shows where identified TSS peaks fall relative to genomic features in the genome assembly CSIRO_AGI_GalMel_v1. *x* axis represents base-2 logarithm of the ratio of observed (obs) counts to expected (exp) counts, and *y* axis shows the genomic features annotated in the genome assembly CSIRO_AGI_GalMel_v1. The defined TSS peaks show a strong and highly significant enrichment at annotated promoters as well as marked depletion in intronic and intergenic regions.

We further examined dinucleotide composition at dominant CTSSs (Figure 3D). This analysis revealed strong enrichment of canonical promoter-associated dinucleotides at the −1/+1 position, consistent with the initiator (Inr) sequence preferences of RNA polymerase II promoters in insects, in which the primary Inr consensus has been described as TCAKTY.^38^ These findings further support the accuracy of the identified promoter-associated CAGE signals.

### Cluster peaks with the highest pooled CTSSs signal overlapping the 5′ end UTRs

We tested for the overlap of the identified TSS peaks and the 5′ end. Peaks ranked by pooled TPM show progressive increase in the 5′ end annotated peaks toward the high expression bins, while the rest of the features (CDS, intron, intergenic, and 3′ end) are lined in low expression bins (Figure 3E). These results are consistent with the canonical promoter activity and indicate the relationship between expression level and the genomic context of the identified CAGE peaks. We conducted genome-wide enrichment analysis to examine the representation of the CAGE signals in the *G. mellonella* gene model. The enrichment analysis of the retained CAGE signals showed strong and specific enrichment at promoter/TSS regions for promoter-associated biotypes (mRNA promoters and transcript promoters) (+ log_2_[observed/expected]). In contrast, intergenic and intronic regions were depleted, with negative log_2_(observed/expected) values, while transcription termination sites (TTS) showed enrichment levels close to background. Likewise, enrichment was also observed at promoters of non-coding RNA classes (lncRNA, snoRNA, snRNA, and tRNA), reflecting accurate capture of diverse transcription initiation events (Figure 3F). Together, these results validated the quality of the CAGE data and demonstrated that highly expressed CTSSs captured canonical promoter architecture.

### Identified TSSs and active enhancers confirm known annotation and expression patterns

We annotated the merged set of TSS and enhancer clusters (28,083 total) using TxDB genomic feature classes. Approximately 30% of TSS clusters overlapped annotated promoter regions, whereas enhancer clusters mapped exclusively to intronic and intergenic regions (Figure 4A). Promoter-associated TSS clusters were the most highly expressed, while non-promoter TSS clusters generally showed low expression, with the exception of clusters annotated to 5′ UTRs and exons. Enhancer clusters annotated to intronic and intergenic regions were consistently lowly expressed relative to TSS clusters (Figure 4B).

**Figure 4:**
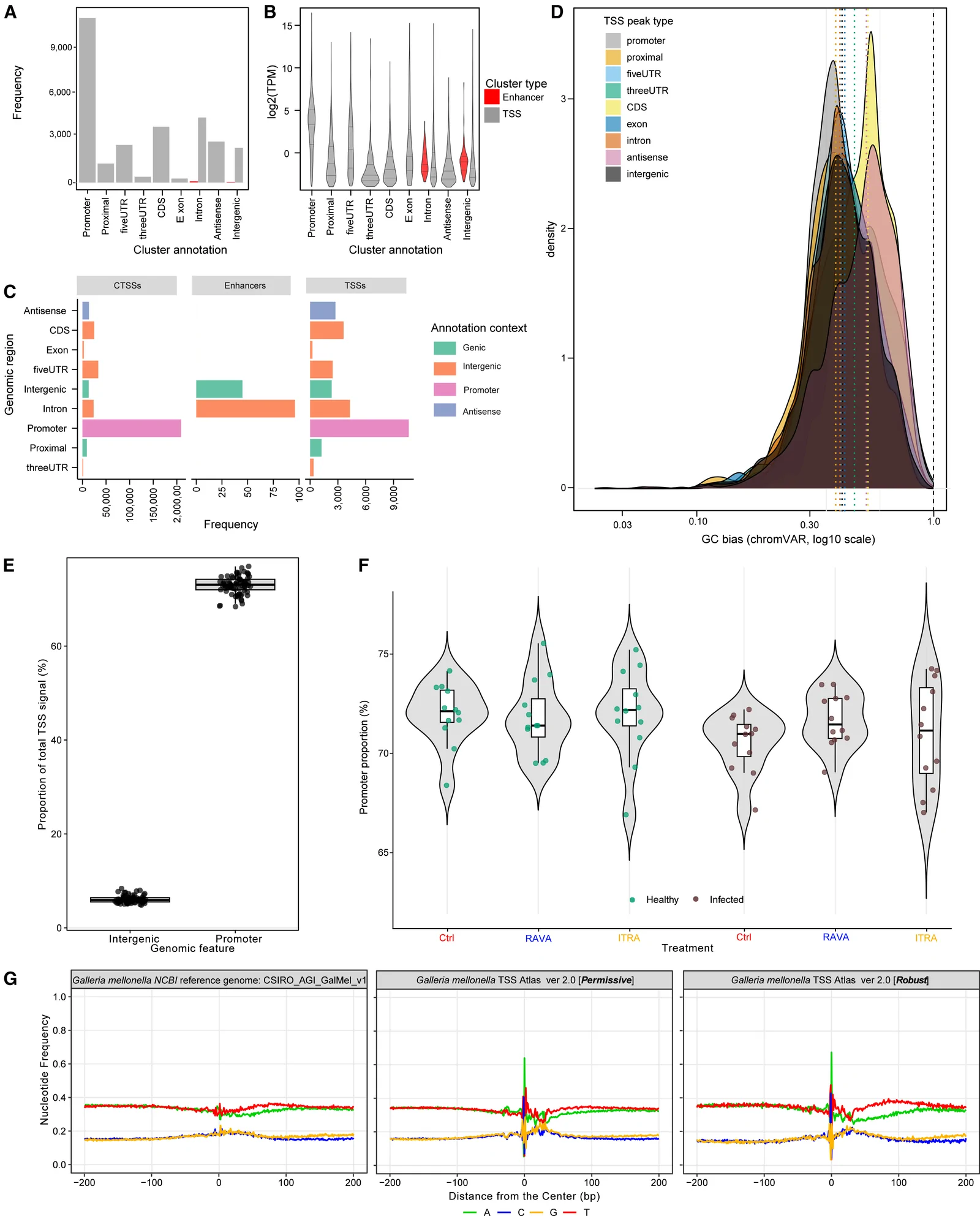
Genomic annotation of LQ-ssCAGE defined TSS and genome-wide nucleotide frequency at TSSs (A) Annotation of the cluster by genomic feature categories. TSS peaks are annotated using CSIRO_AGI_GalMel_v1 genomic features. The majority (36.1%) of defined TSS clusters are annotated as promoters. (B) Gene expression per cluster annotation. As expected, clusters overlapping promoters have higher gene expression values (log_2_ TPM) compared to enhancers. (C) Annotation by the genomic context of CTSSs, enhancers, and TSSs. The majority of CTSS and TSS peaks overlap promoter regions, while enhancer peaks overlap genic and intergenic regions. (D) GC bias distribution of TSS regions obtained by chromVAR function. The peak shows that the promoter TSS peak type has a high GC bias. (E) Promoter signal proportion by condition and treatment. (F) Per-library promoter and intergenic signal proportions. Each violin represents a treatment condition or untreated control. Samples are grouped into healthy and infected categories. Boxplots indicate the median and interquartile range, and points represent individual libraries, colored by infection status. (G) Three plots of the nucleotide frequency relative to annotated gene 5′ ends. The current gene annotation of the *G. mellonella* reference genome provided by NCBI (left). New annotation of the *G. mellonella* atlas version 2.0 (permissive set, middle) and the *G. mellonella* atlas version 2.0 (robust set, right). Version 2.0 shows a clear improvement of the annotation with strong core promoter sequence bias (TATA/Inr/GC patch).

To further validate these assignments, we classified CTSSs, TSSs, and enhancer clusters by genomic context. Promoters represented the largest category, accounting for 55.6% of filtered CTSSs (Figure 4C). The remaining CTSSs were mapped to CDS, intronic, 5′ UTR, antisense, proximal, 3′ UTR, and exonic regions. TSS peaks were likewise predominantly promoter associated, whereas enhancer clusters were restricted to genic and intergenic regions (Figure 4C). Analysis of sequence composition showed that promoter-associated TSS peaks had the strongest GC bias (Figure 4D), consistent with the CpG-rich architecture of canonical promoters. In contrast, intergenic and intronic TSS peaks displayed reduced GC bias, consistent with enhancer-like initiation patterns.

We next asked whether infection or antifungal treatment altered TSS signal distribution using an established *M. mycetomatis*-infected *G. mellonella* model profiled by RNA-seq and LQ-ssCAGE. RNA-seq identified differentially expressed genes between infected and healthy larvae, consistent with our previous findings.^1^ By contrast, no differentially expressed genes were detected between infected larvae and infected larvae treated with itraconazole or ravuconazole. This is in line with recent clinical observations showing no measurable activity of either drug during the first 6 months of treatment, as evidenced by persistent fungal viability and the absence of reductions in lesion size or plasma β1,3-D-glucan levels.^33^^,^^39^

We then quantified the fraction of total TPM arising from promoter and intergenic regions across all 72 libraries (Figure 4E) and visualized the proportions by condition and treatment group (healthy or infected; no treatment, ravuconazole, or itraconazole) (Figure 4F). Representative genome browser views illustrate the distinct signal architectures of TSS and enhancer clusters (Figures S5A and S5B). TSS clusters showed the expected unidirectional pattern, whereas enhancer clusters displayed the bi-directional signal characteristic of active enhancers. Annotated TSS peaks (permissive and robust) are provided in GTF files.

Finally, to assess annotation quality, we plotted nucleotide frequency around annotated gene 5′ ends for three annotations: the *G. mellonella* NCBI RefSeq reference genome (CSIRO_AGI_GalMel_v1), the *G. mellonella* TSS Atlas v2.0 permissive set, and the v2.0 robust set (Figure 4G). Both atlas-derived annotations showed a markedly stronger core promoter sequence signature than the NCBI reference annotation, supporting improved resolution and accuracy of TSS annotation in the atlas.

### Identified broad and sharp promoters confirm known features

Promoters identified by CAGE analysis have been classified into two categories^40^: broad or sharp promoters. To classify TSSs, we first calculated the interquartile range (IQR). The distribution of IQRs showed that most of the TSSs were above or below 10 bp (IQR) (Figure 5A). We used 10 bp IQR as the cut-off value to classify the TSSs into broad or sharp promoters. The classification resulted in 4, 746 broad promoters and 1,679 sharp promoters. Broad and sharp promoters are known to have distinct features, including CpG island enrichment, TATA-box motifs, and other regulatory elements. We tested the core promoter sequences of the broad and sharp TSS peak −40/+10 bp and plot their sequences logo. Compared to broad promoter, sharp TSSs have a stronger TATA-box upstream of the TSS (Figure 5B). We further analyzed the density of GC content distribution across the promoter class (sharp and broad) and positional enrichment of TATA box (Figure 5C). The broad distribution of GC contents (%) indicates GC-rich promoters associated with CpG island. Enrichment of TATA box confirms the occurrence of the TATA box ∼30 bp upstream of the TSS, the canonical TATA box position. Figure 5D demonstrates two examples of broad and sharp promoters. The broad promoter “NW_026441966.1:42202–42341;+” had multiple spaced TSSs, while the sharp promoter “NW_026441966.1:2691787–2692193;+” had a single well-defined TSS as described. The broad promoter “NW_026441966.1:42202–42341;+” has the transcript type of promoter and overlap the protein-coding gene Tpst—tyrosylprotein sulfotransferase (LOC113510420). In *G. mellonella*, Tpst has not been studied before, but in *D. melanogaster*, studies show its role as a post-translational modification enzyme, which has been conserved across species.^41^^,^^42^^,^^43^ The sharp promoter “NW_026441966.1:2691787–2692193;+” overlapped the protein-coding gene Acyl-CoA-binding proteins (ACBPs) (LOC113515269). ACBPs are multifunctional proteins and are highly conserved in insects.^41^ ACBPs play roles in several BPs including lipid metabolism, fatty acids transport, and signaling. Studies on *Drosophila* show that multiple ACBP genes exist, and different isoforms were involved in different BPs and egg development.^44^

**Figure 5:**
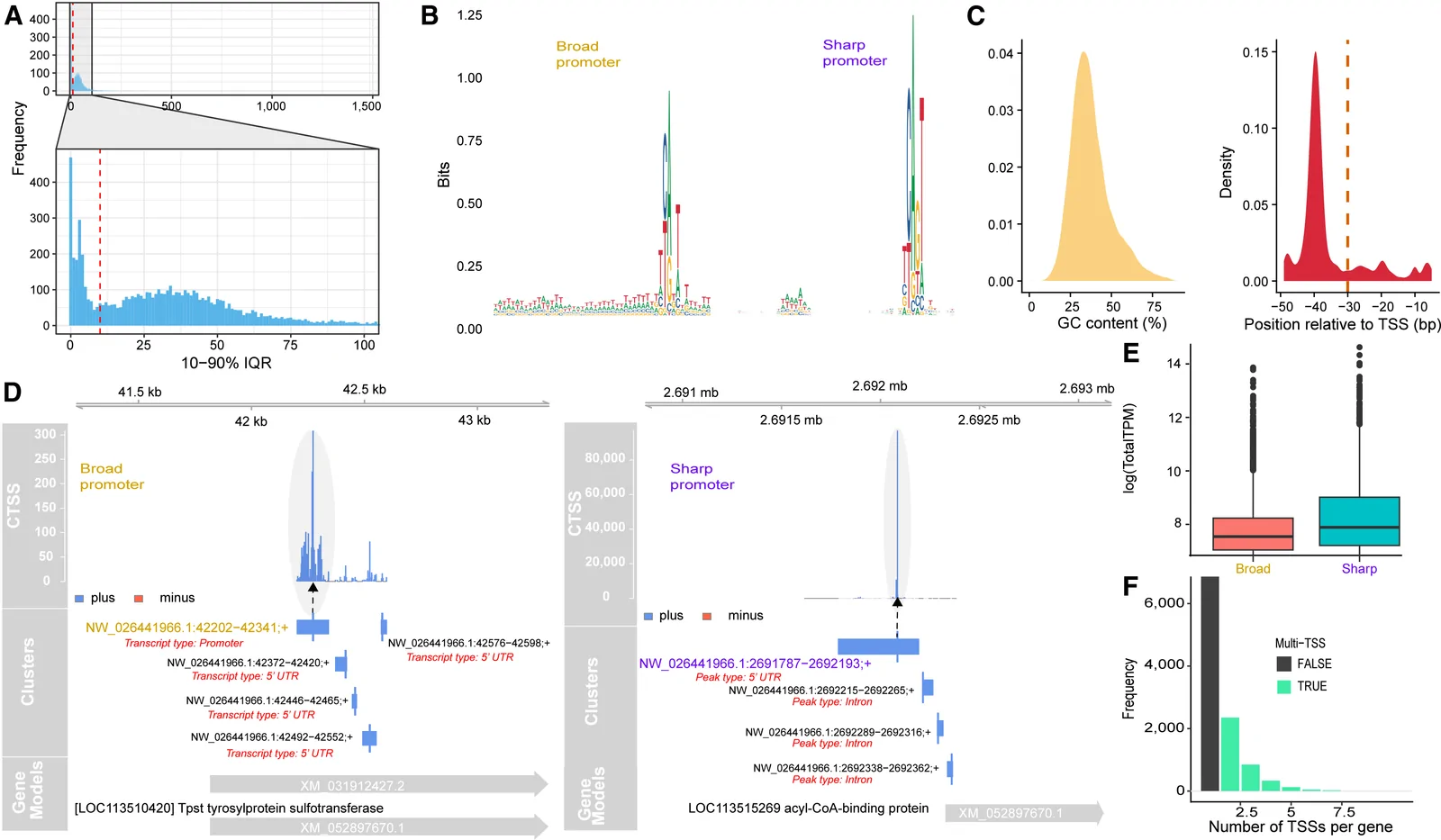
Broad vs. sharp promoter features and TSS gene usage (A) Identification of broad and sharp promoter. The bar plot shows the distribution (bi-modal) of the interquartile range (IQR). IQR divides the TSS into sharp and broad TSSs. The dashed line indicates that TSSs are either below or above an IQR of ten. Broad promoters have IQRs below ten, and sharp promoters have IQRs above 90. (B) TATA-box and CpG island enrichment analysis. The Seqlogo for the broad promoter enriched for GC content in the broad promoter (left) and the enrichment of TATA-box upstream of the TSS peak of the sharp promoter indicates core promoter features. (C) GC content distribution (left) the density distribution plot shows the GC contents (%) across the promoter class (sharp and broad). The broad distribution indicates GC-rich promoters associated with CpG island. Positional enrichment of TATA box (right) shows the occurrence of the TATA box ∼30 bp upstream of the TSS indicated by the dashed red line (the canonical TATA box position). (D) Example of a broad promoter (left panel) with multiple spaced TSS clusters, and a sharp promoter (right) with a single TSS. Annotated genes transcripts within each promoter are shown in the gene model panel. (E) Distribution of expression between broad and sharp promoters shows a slight difference in the normalized expression between the two classes of promoters. (F) TSS usage by gene. Differential TSS usage tests whether a gene uses alternative TSSs under different conditions. The figure indicates that the majority of (∼6,899) *G. Mellonella* genes utilize a single TSS, and ∼2,300 genes utilize multiple TSSs.

We determined the expression difference between broad and sharp promoters, we observed slight differences in the expression between the two categories (broad and sharp) (Figure 5E). Next, we looked at differential TSS usage (DTU). DTU aims to analyze if a gene uses different TSSs under different conditions, analogous to alternative splicing identification in RNA sequencing.^45^ Our analysis of the TSS usage indicated that most of the genes use a single TSS, while only a few genes use more than one TSS to initiate the expression of the gene (Figure 5F). To further explore whether infection affects promoter choice, we analyzed DTU between healthy and infected samples. Consistent with our global TSS usage analysis, most expressed *G. mellonella* genes were associated with a single dominant TSS, whereas a subset of genes showed evidence of multiple TSS usage. DTU analysis identified several genes with significant condition-associated changes in the relative contribution of alternative TSSs (Figure S6). Among the top-ranked genes, LOC113511966 (Cystinosin homolog) and LOC113518960 (Peroxidase-like isoform X3) showed clear shifts in TSS usage between healthy and infected samples. These results suggest that infection may influence promoter selection and alternative TSS usage in *G. mellonella*, providing an additional layer of transcriptional regulation beyond changes in overall gene expression. To explore whether genes with DTU were associated with specific biological functions, we performed GO enrichment analysis using the ranked list of DTU-associated genes (Figure S7). Several GO terms related to membrane-associated processes, transporter activity, ion binding, enzymatic activity, and metabolic functions were observed among the top-ranked terms. However, none of the displayed GO terms passed the multiple-testing correction threshold of false discovery rate (FDR) <0.05. These results suggest that DTU was not restricted to a single significantly enriched functional category. The analysis should therefore be interpreted as exploratory, while also reflecting the current limitations of functional annotation in *G. mellonella*.

### TSS-enhancer interactions

CAGE data can be used to predict physical interaction between enhancers and TSSs,^34^ thereby identifying the genes that are regulated by enhancers.

We calculated pairwise correlations in expression (TPM) between TSSs and enhancers within a distance of 50 bp with Kendall’s tau. Considering a positive correlation and a *p value* < 0.05, we found that 682/37,320 TSSs are interacting with 156/218 active enhancers. Figure 6A shows that the enhancer “NW_026442130.1:2231109–2231625” has interactions with three TSSs as shown by the links track. The distal TSS “NW_026442130.1:2238096–2238234;+” has the highest correlation upstream of the gene ER membrane protein complex subunit 7 (LOC113521644) with *p* = 0.01). We compared the average CAGE expression between interacting and non-interacting TSSs and observed that the interacting TSSs have higher average gene expression (Figure 6B).

**Figure 6:**
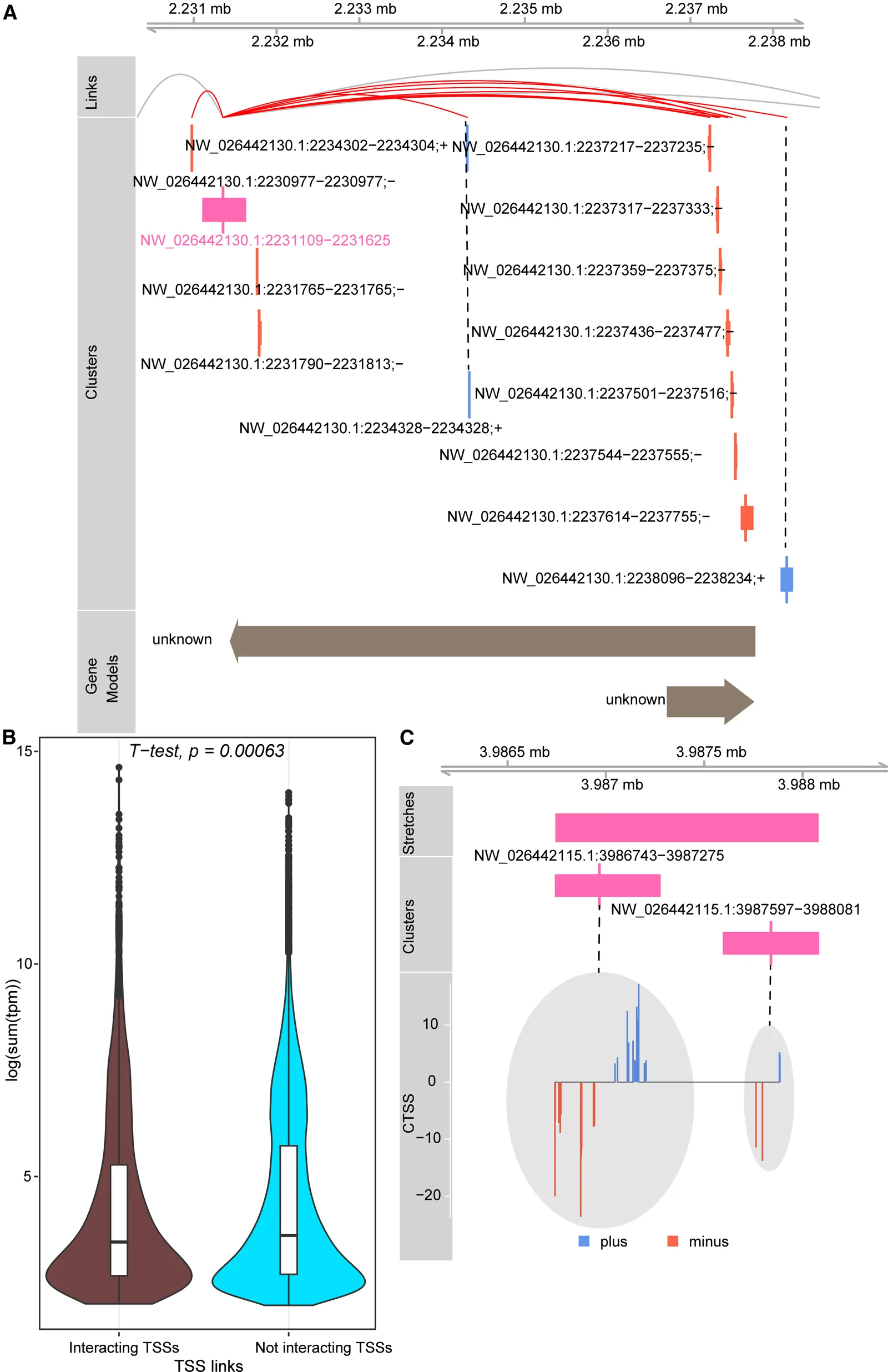
TSS-enhancer interactions and stretched enhancers (A) Genome browser view of interacting TSSs and enhancers. The genome view consists of three tracks: links, identified clusters, and pooled CTSS signals. By using the distance and expression correlation patterns between TSSs and enhancers, we predict enhancers-TSSs links. The links track shows the strength of correlation between 8 TSSs and the NW_026442130.1:2231109–2231625 enhancer. The highest correlation was found between NW_022270367.1:4182931–4183061;+ TSS and the enhancer (dashed orange line). (B) Variations in the gene expression between interacting and non-interacting TSSs. The violin plot shows that TSSs interacting with enhancers is highly expressed compared with the non-interacting TSSs. (C) Genome browser view of super-enhancers. Super or stretch enhancers are closely spaced enhancers. The figure shows two stretched enhancers with bi-directional expressions. The stretches are visualized by the stretches track on top of the genome browser view.

### Identification of super enhancers

Super-enhancers (stretch enhancers) are “groups of putative enhancers in close genomic proximity with unusually high levels of mediator binding.”^46^ We identified the stretches by grouping nearby proximal enhancers into groups, each consisting of a cluster of enhancers. After identifying the nearby groups, an average pairwise correlation between them was calculated. In our data, we identified a set of stretches; Figure 6C shows the first stretch with three bi-directional enhancers overlapping intergenic regions.

### Homer *de novo* motif for *G. mellonella* and TF expression analysis

We aimed to identify enriched motifs and their sequence patterns that appear more frequently than expected by chance in the set of the identified TSS peaks for *G. mellonella*. HOMER^47^ command findMotifsGenome.pl was used to predict the enriched motifs and corresponding TFs for *G. mellonella* in the top 5% of the most highly expressed TSS based on the CAGE signal (1,866: 37,320). HOMER predicted two types of TFs: HOMER *de novo* motif and HOMER known motif enrichment. *De novo* motif discovery identified significantly multiple enriched sequence motifs within the analyzed regions (Figure 7A) (data and code availability). The most strongly enriched motif corresponded to an RFX family transcription factor binding (JASPAR MA0365.2; similarity score = 0.827). For each of the identified motifs, we obtained the TF from the Jaspar database^48^; since *G. mellonella* was not supported by Jaspar yet, we obtained the ortholog for each TF using NCBI blastx.^49^ Several of the identified TFs have the ortholog *G. mellonella* genes. Using HOMER motif enrichment results, we computed enrichment and ranked the TF motifs by their computed enrichment score (Figure 7B). Our motif enrichment analysis identifies significant (7.1-fold enrichment) overrepresentation of the TF RFX1 (LOC113510972). RFX1 belongs to fork head/winged helix factors class and is conserved across animals, including insects. In *D*. *melanogaster* (FBgn0020379), RFX is a key regulator of ciliary, which binds the X-box motif and sensory neuron gene expression. The gene expression of the top-enriched TF was shown in the heatmap (Figure 7C). LQ-ssCAGE gene expression of the predicted TFs shows a difference in gene expression between healthy and infected samples (broadly expressed).

**Figure 7:**
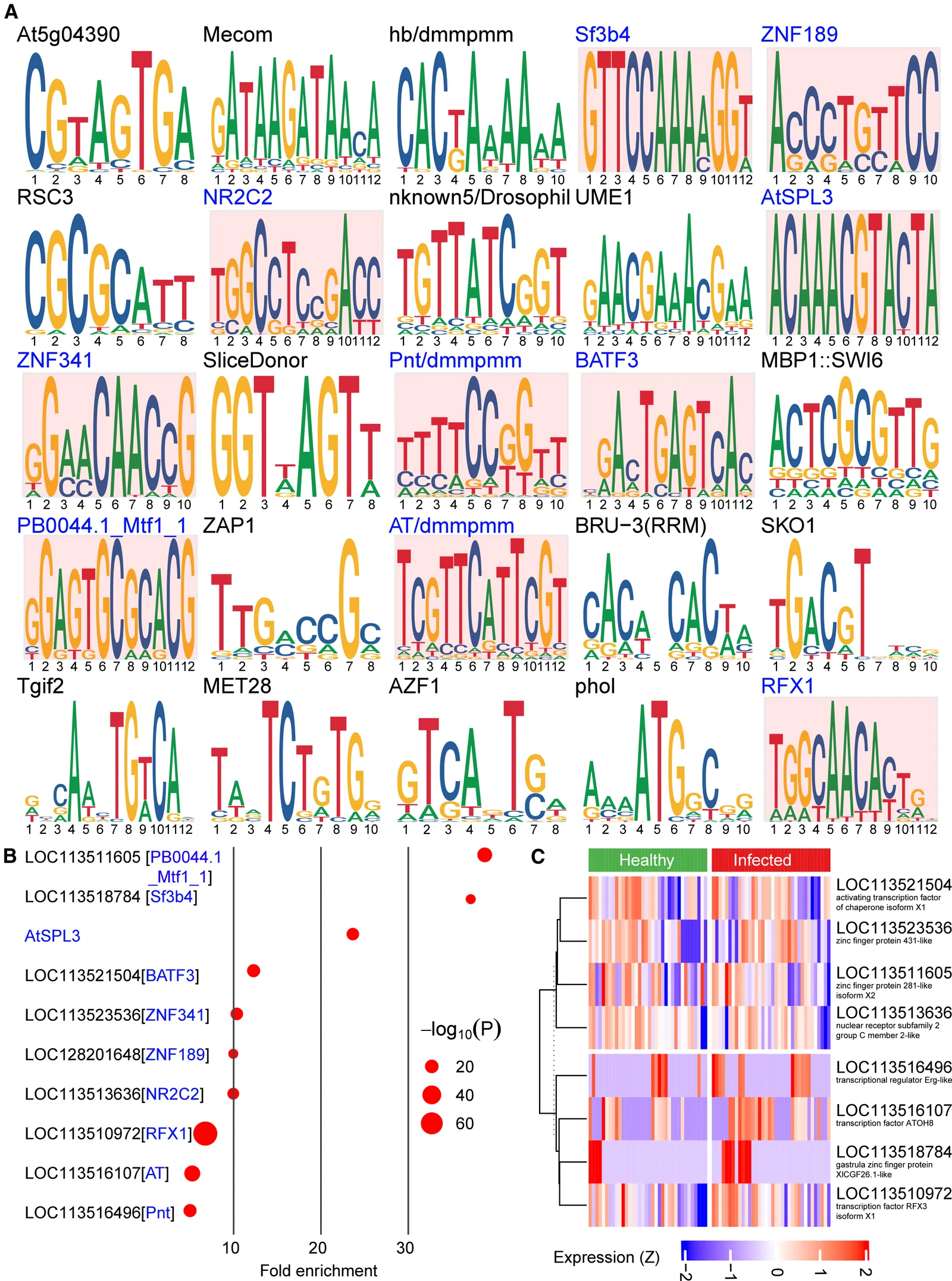
Motifs prediction and analysis (A) TF motif sequence logos. The 5% highly express TSS peaks is used as input for HOMER suite for *de novo* motif prediction command. The figure shows the seqlogos of the top predicted motifs and their corresponding transcription factor expression. The TF plotted inside a red box indicated the *G. mellonella* ortholog each boxplot shows the CAGE expression as TPM for the newly identified top TFs across samples between infected and healthy *G. Mellonella*. (B) TF enrichment. The figure shows the fold enrichment of the ortholog gene of *G. mellonella*. (C) Gene expression of the enriched *G. mellonella* TFs represented as heatmap. The samples are annotated by their status (healthy/infected).

### Genomic views of the *G. mellonella* annotation on ZENBU genome browser

Genome browsers are a useful tool to visualize and explore genomic features in a 2D view using genomic coordinates of the genes (features). To understand the localization of the mapped TSS peaks and identified enhancers, we first uploaded the genome assembly CSIRO_AGI_GalMel_v1 to the ZENBU genome browser. All annotated features in CSIRO_AGI_GalMel_v1 are searchable and can be explored (e.g., zoom-in or zoom-out). On top of the genome assembly, the LQ-ssCAGE mapped reads are also uploaded to the genome browser. Together with mapped CAGE tags (in BAM format), detailed metadata about each sample is uploaded as well. The metadata, together with mapped CAGE tags, allows grouping of the sample experiments per treatment (time point, replication, etc.) and makes for a more interesting ZENBU genome browser view.

Based on our experimental design, we created four tracks with different groupings based on the experiment’s conditions (https://fantom.gsc.riken.jp/zenbu/gLyphs/#config=Galleria_mellonella_TSS).

## Discussion

The lack of TSS information on the invertebrate *G. mellonella* genome will limit its utilization as an experimental model in the wider research community. To address this, we performed genome-wide mapping and analysis of TSSs in healthy and infected *G. mellonella* larvae (with and without azole treatment) using LQ-ssCAGE.

We obtained high consistency in sequencing depth of all libraries with expected gene body coverage across all samples. Pairwise comparison of the mapped 5′ end tags and RNA-seq reads shows a high correlation between the two methods and confirms the reproducibility of the expression profiles by two independent sequencing methods, as reported by Kawaji et al.^50^

LQ-ssCAGE allowed us to map 1,219,124 CTSSs for *G. mellonella* larvae. The quality of the resulting CTSS was confirmed by genomic annotation and GC bias. The annotation of the CTSSs confirmed a promoter-rich CAGE profile, and the analysis of GC bias confirmed a highly elevated GC level. TSS regions annotated as promoters are highly expressed compared to enhancers; the variation of the expression patterns between promoters and enhancers here lines up with previously reported results.^51^

From the CTSS profile, we identified 27,942 permissive TSS peaks and 5,437 robust TSS peaks. In addition, we identified a total of 728 enhancers. We assigned genomic annotations to the identified TSS peaks and enhancer regions.

The promoter shape plays a fundamental role in the gene expression regulation in animals. We investigated promoter architecture; our analysis revealed the presence of both sharp and broad promoters. This is consistent with conserved features and characteristics of the CAGE data and eukaryotic transcription initiation. Sharp promoters were associated with higher positional precision of TSSs and stronger sequence motif enrichment (TATA box), whereas broad promoters displayed dispersed initiation patterns and elevated GC content.

In addition to the promoter shape, we analyzed the differentiation of TSS usage (i.e., genes use different numbers of TSSs under different conditions).^29^ We show that in *G. mellonella*, most of the genes use a single TSS, but some genes use more than one TSS to initiate expression.

One of the features of CAGE is that it enables the prediction of physical interaction between enhancers and TSSs. This kind of interaction indicates which gene is regulated by which enhancer. As reported previously, TSSs and enhancers in proximity have highly correlated gene expression and are potentially interacting.^34^ We identified a set of interactions between TSS and enhancers. This enabled us to classify the TSSs as either interacting TSSs or non-interacting TSSs. We observed quite different gene expression between the two classes of TSSs.

As reported by Pot and Lieb,^46^ super-enhancers (stretched enhancers) demonstrate chromatin characteristics and function differently from singleton enhancers. In our LQ-ssCAGE data, we detected two stretches, each with 3 clusters of enhancers (9 super enhancers).

To compare our TSS annotation of *G. mellonella*, we investigated the availability of TSS annotations for closely related species. First, we considered *Bombyx mori* (NCBI taxonomy: 7091) from the family Lepidoptera and same clade (Obtectomera) as *G. mellonella*. In SilkBase,^52^ the only TSS set was obtained computationally by mapping the BmN4-derived TSS tags to the *B. mori* genome,^53^ with no characterization of TSS and promoters.

The results of the several QC tests and the genome-wide enrichment analysis results (Figure 3F) demonstrate high specificity of CAGE tags for TSSs and confirm the genome-wide quality and biological relevance of the identified TSS atlas.

Our results contribute to the genome annotation of *G. mellonella* and will enable the study of gene regulation research in this unique model. The raw sequence reads, and processed CAGE data are available via NCBI GEO^54^ and ZENBU genome browser.^55^ The resulting TSS atlas in this study will be a centerpiece for the development of a reference TSS for the *G. mellonella like the* human and mouse refTSS we developed previously (https://reftss.riken.jp/).^12^

### Limitations of the study

We identified only a small number of enhancers, likely reflecting both technical and biological constraints of the dataset. LQ-ssCAGE is not optimized to capture nascent transcription, enhancer RNAs (eRNAs), or weak intergenic enhancer-associated signals, and therefore has limited sensitivity for enhancer discovery. In addition, the sequencing depth used here (∼700,000 reads per sample) may have further reduced the detection of low-abundance regulatory elements.

To assess the effect of sequencing depth on TC discovery, we performed saturation analysis (Figure S8A). The fixed consensus TC saturation curve showed a rapid increase in detected TCs at low depth, indicating efficient recovery of highly expressed promoters, followed by a marked reduction in the rate of TC discovery as depth increased. Beyond ∼2 × 10^5^ reads, additional sequencing yielded progressively fewer new TCs, and the curve approached a plateau at ∼6 × 10^5^ to 7 × 10^5^ reads, consistent with near-saturation of TC detection under the current framework.

We also examined whether TC recovery was driven by sequencing depth or by biological condition. Comparison of healthy and infected samples (Figure S8B) indicated that overall TC detection was determined primarily by read depth, with little effect of experimental condition at the global level. Together, these results suggest that the current sequencing depth was sufficient to recover most highly expressed TCs, but likely underestimated weakly expressed TCs and enhancer-associated transcription. Thus, the limited number of enhancers identified in this study likely reflects the combined effects of assay design and sequencing depth rather than a true paucity of enhancer activity in *G. mellonella*.

## Resource availability

### Lead contact

Requests for further information and resources should be directed to and will be fulfilled by the lead contact, Imad Abugessaisa (imad.abugessaisa@a.riken.jp).

### Materials availability

This study did not generate new unique reagents.

### Data and code availability

• LQ-ssCAGE raw data have been deposited at GEO at GSE282923 and are publicly available as of the date of publication.
• Processed LQ-ssCAGE data including aligned reads in BAM format, CTSS, TCs, the BDgenome, and annotation R Bioconductor packages for *Galleria mellonella* have been deposited to Mendeley Data at https://doi.org/10.17632/hg9ymt3px9.6 and are publicly available as of the date of publication.
• All original code has been deposited at Mendeley Data at https://doi.org/10.17632/hg9ymt3px9.6 and is publicly available as of the date of publication.
• ZENBU genome browser views can be accessed through the link below https://fantom.gsc.riken.jp/zenbu/gLyphs/#config=Galleria_mellonella_TSS.
• All original code has been deposited at Mendeley Data at https://doi.org/10.17632/hg9ymt3px9.6 and is publicly available as of the date of publication.
• Any additional information required to reanalyze the data reported in this paper is available from the lead contact upon request.
• Resulting GTF files generated in this study are have been deposited to Mendeley Data under the GTFs folder at https://data.mendeley.com/datasets/hg9ymt3px9/6.

## Acknowledgments

We thank Atsui Hiroto, Nana Tamura, Nobuyuki Takeda, Teruaki Kitakura, Akira Furukawa, Dounia El Yachioui, and Ulugbek Yakhshimurodov for providing technical and administrative support. We thank RIKEN Center for Integrative Medical Sciences sequence platform for their helpful support in sequencing RNA. We thank the Drugs for Neglected Diseases initiative (Geneva) for support of MycetOS. We would also like to thank Scott Walker from RIKEN Center for Integrative Medical Sciences for English proofreading. This work was supported by research grants from World Premier International Research Center Initiative (WPI); Japanese Ministry of Education, Culture, Sports, Science and Technology (10.13039/501100001700MEXT), Japan; the Dutch Research Council by Aspasia grant no. 015.013.033 and VICI grant no. 09150182310010; and the Erasmus University with 10.13039/501100001828EUR Fellowship, all three awarded to W.W.J.v.d.S. This study is also supported by The 10.13039/501100001691Japan Society for the Promotion of Science, 10.13039/501100001691KAKENHI grant no. 20K08832 awarded to I.A. Research Grants from the Japanese Ministry of Education, Culture, Sports, Science and Technology (MEXT) were provided to 10.13039/501100006264RIKEN Center for Integrative Medical Sciences.

## Author contributions

LQ-ssCAGE experiments, R.I.M., T.K., and H.K.; RNA-seq experiments, S.N.; RNA-seq and LQ-ssCAGE raw data processing, M.T., A.H., and I.A.; sequence data management, C.T.; administration of LQ-ssCAGE and RNA-seq experiments, Y.O.; writing – original draft, I.A., M.K., R.I.M., and M.T.; *G. mellonella* sample preparation and RNA extraction, M.K.; creation of ZENBU genome browser views, J.S.; analysis of LQ-ssCAGE, I.A.; results interpretation, I.A. and T.K.; visualization, J.S. and I.A.; fund acquisition, I.A., WdS., and T.K.; project supervision, I.A., W.W.J.v.d.S., and T.K.

## Declaration of interests

The authors declare no competing interests related to this article.

## Declaration of generative AI and AI-assisted technologies in the writing process

During the preparation of this work, the authors used Chatly-Gemini 3 Pro to improve R scripts and understand some of the reviewers’ comments. After using this tool or service, the authors reviewed and edited the content as needed and take full responsibility for the content of the publication.

## STAR★Methods

### Key resources table

**Table:** 

REAGENT or RESOURCE	SOURCE	IDENTIFIER
**Critical commercial assays**		

RNeasy Mini Kit	Qiagen, Germany	74104
RPMI 1640 Medium	Gibco, ThermoFisher, the Netherlands	32404
L-Glutamine (200 mM)	Gibco, ThermoFisher, the Netherlands	25030-024
1.98 mM 4-morpholinepropanesulfonic acid (MOPS)	Sigma-Aldrich, the Netherlands	M9027
100 mg/L chloramphenicol	Oxoid, UK	SR0078H
Soniprep 150 plus, MSE, The United Kingdom	MSE, UK	N/A
Vacuum filtration (Nalgene, Abcoude	Nalgene, Sigma-Aldrich, the Netherlands	Z358193
Phosphate-buffered saline (PBS)	Analytichem, the Netherlands	K283F100CR
Insulin 29G U-100 needle	BD Diagnostics, USA	324891
Itraconazole	Janssen Pharmaceutical Products, Beerse, Belgium	N/A
Ravuconazole	Eisai Co. Ltd.	N/A
Dynabeads™ Oligo(dT)_25_	Invitrogen	61002
MGIEasy RNA Directional Library Prep Set	MGI Tech	1000006386
D5000 Reagent kit	Agilent Technologies	5067-5589
High Sensitivity DNA assay kit	Agilent Technologies	5067-4626
KAPAL ibrary Quantification Kit for Illumina	Kapa Biosystems	KK4828
NanoDrop2000	Thermo Scientific	ND-2000
Qubit dsDNA HS Assay kit	Thermo Scientific	Q32854
DNBSEQ-G400RS	MGI Tech	DNBSEQ-G400RS
DNBSEQ-G400RS High-throughput Rapid Sequencing Kit (FCL PE100)	MGI Tech	1000016950

**Deposited data**		

Genome-wide mapping of the Galleria mellonella larvae transcription start sites during infection with the m. mycetomatis pathogen	This study	GSE282923

**Experimental models: Cell lines**		

N/A	N/A	Wasmotten Forelshop.be

**Experimental models: Organisms/strains**		

*Galleria mellonella* larvae Greater wax moth	Forelshop (Tremelo, Belgium) https://www.forelshop.be/levend-aas-big-big-sagip/wasmotten-forelshopbe	Wasmotten Forelshop.be
*Madurella mycetomatis*	Erasmus MC biobank	mm55

**Oligonucleotides**		

oligo dT beads	Included in the library or sequencing kit	N/A
Random hexamers	Included in the library or sequencing kit	N/A

**Software and algorithms**		

Bioconductor	Gentleman et al.^56^	https://www.bioconductor.org/
Gviz	Hahne F et al.^57^	https://doi.org/10.18129/B9.bioc.Gviz
chromVAR	Schep et al.^58^	https://doi.org/10.18129/B9.bioc.chromVAR
CAGEfightR	Thodberg et al.^29^	https://bioconductor.org/packages/release/bioc/html/CAGEfightR.html
Bedtools	Quinlan et al.^59^	https://bedtools.readthedocs.io/en/latest/
Samtools	Li et al.^60^	https://www.htslib.org/doc/samtools-reference.html
STAR: RNA-seq aligner	Dobin et al.^61^	https://github.com/alexdobin/STAR
SkewC	Abugessaisa et al.^62^	https://github.com/LSBDT/SkewC
HOMER	Heinz et al.^63^	http://homer.ucsd.edu/homer/
AnnotationForge	Carlson^64^	https://bioconductor.org/packages/AnnotationForge.
FastQC	Wingett et al.^65^	https://www.bioinformatics.babraham.ac.uk/projects/fastqc/
MOIROAI	Hasegawa et al.^36^	N/A
R Statistical	R Core Team (2025)	https://cran.r-project.org/index.html
Genome assembly CSIRO_AGI_GalMel_v1	NCBI	GCF_026898425.1
blastx	NCBI	https://blast.ncbi.nlm.nih.gov/Blast.cgi
BSgenomeForge	Pagès and Kakopo^66^	https://bioconductor.org/packages/BSgenomeForge.

**Other**		

4200 TapeStation system	Agilent Technologies	G2991BA
D5000 ScreenTape	Agilent Technologies	5067–5588
2100 Bioanalyzer	Agilent Technologies	G2939BA
Qubit 3.0 Fluorometer	Thermo Scientific	Q33216
HiSeq2500	Illumina	SY-401-2501

### Experimental model and study participant details

• Species Galleria mellonella.

 Taxonomy ID:NCBI:txid7137

 GenBank common name: greater wax moth

 NCBI BLAST name: moths & butterflies

 Rank: species

 Development stage: Larvae

 *G. mellonella* larvae were obtained from Forelshop (Tremelo, Belgium) and kept in the dark on wood shavings at room temperature until use. Within five days of receipt, larvae of approximately 300–500 mg were selected for experimental use. The selected larvae were divided into Petri dishes containing 90 mm Whatman filter paper and five larvae per dish.

• Species M. mycetomatis

 Strain MM55

 Taxonomy ID:NCBI:txid 100816

 NCBI BLAST name: ascomycete fungi

 Rank: species

 An inoculum of *M. mycetomatis* was prepared following the method described by Kloezen et al.^4^

### Method details

#### *G. mellonella* acquisition and infection

An inoculum of *M. mycetomatis* strain MM55 was prepared following the method described by Kloezen et al.^4^ In short, the mycelium was harvested, transferred to RPMI-media supplemented with 0.35 g/L L-glutamine, 1.98 mM 4-morpholinepropanesulfonic acid (MOPS) and 100 mg/L chloramphenicol, sonicated for 30 s at 10 microns (Soniprep 150 plus, MSE, The United Kingdom) and incubated for two weeks at 37°C. Next, the mycelium was harvested using vacuum filtration (Nalgene, Abcoude, the Netherlands, using a 22-micron filter (Whatman)). The fungal biomass was scraped from the filter, and the wet weight was measured before resuspending the mycelium in phosphate-buffered saline (PBS). The suspension obtained was sonicated for 120 s at 10 microns, washed in PBS, and diluted to a final concentration of 4 mg per 40 μL. The larvae were infected using the prepared inoculum by injection in the last left proleg using an insulin 29G U-100 needle (BD Diagnostics, Sparsk, USA). After 4, 30, and 52 h post-infection, larvae were treated with itraconazole or ravuconazole to a final concentration of 5.71 mg/kg, a clinically relevant dose of 400 mg based on an average human with a weight of 70 kg.^67^ The course of the infection was monitored by infecting and treating separate groups consisting of 15 larvae per group, and survival was recorded daily for ten days. During all experiments, Pupae were removed from the equation, and non-infected larvae were included as a control.

#### RNA isolation

RNA was isolated at 6-, 30-, 72- and 168 h post-infection. The contents of three larvae were pooled, flash-frozen with liquid nitrogen, and mechanically crushed using a pestle and mortar. The resulting powder was suspended in RLT buffer (supplemented with 1% β-Mercaptoethanol), provided in the RNeasy Mini Kit (Qiagen, Germany). The samples were incubated at 57°C for 3 min before proceeding according to the manufacturer’s instructions.

#### RNA-seq library preparation and sequencing

RNA-seq library was prepared using MGIEasy RNA Directional Library Prep Set (MGI Tech) according to the manufacturer’s protocols. We used 1 μg of total RNA for each sample for library preparation. PolyA+ RNA was enriched from the total RNA with oligo dT beads before preparing the RNA-Seq library. Random hexamers were used for the reverse transcription reaction and 14-cycle PCR reaction (Manual’s default setting). Resulting RNA library was QCed using Qubit dsDNA HS Assay Kits (Thermo Fisher Scientific) and Agilent TapeStation, with High Sensitivity D5000 Reagents (Agilent Technologies), and sequenced with 100 bp paired end reads using a DNBSEQ G400 Sequencing platform (MGI Tech).

#### LQ-ssCAGE library preparation and sequencing

LQ-ssCAGE started with Reverse Transcription (RT) of 200 ng of total RNA to generate 5′ end of the capped RNA/first-strand cDNA hybrids. The RNA/cDNA hybrids were cap-trapped and adapted with illumina sequencer-compatible barcode-tagged adaptors.^21^ No PCR amplification was performed during the LQ-ssCAGE library preparation. The number of resulting libraries was checked by qPCR using KAPA Library Quantification Kits (Kapa Biosystems). LQ-ssCAGE library sequenced using Illumina HiSeq2500 platform in Rapid mode with PE read type and 50 base read length.

#### RNA-seq library preparation and sequencing and mapping

RNA-seq library was prepared using MGIEasy RNA Directional Library Prep Set (MGI Tech) according to the manufacturer’s protocols. We used 0.34 to 1 μg of total RNA measured by NanoDrop2000 for each sample for the library preparation. PolyA+ RNA was enriched from the total RNA with oligo dT beads prior to preparing the RNA-Seq library. Resulting RNA library was QCed using Agilent Bioanalyzer with High Sensitivity DNA assay kit (Agilent Technologies) and sequenced with 100 bp PE reads using a DNBSEQ G400 Sequencing platform (MGI Tech) and a DNBSEQ-T7 Sequencing platform (MGI Tech). Fastq files are mapped using STAR aligner (v2.5.3) against the Genome assembly CSIRO_AGI_GalMel_v1

#### LQ-ssCAGE data processing

LQ-ssCAGE PE sequence data provides cDNA sequence as Read1 and sample barcode sequence as Read2. An extra untemplated G at the first base of the sequenced reads were detected and trimmed using in-house script. To demultiplex sample barcode sequence, an in-house script is used with Read1 and Read2 data. Analysis of LQ-ssCAGE tags is performed by Moirai workflow^36^ using Read1 cDNA read (Figure S4). The genome mapping software used was STAR (v2.5.3) against the Genome assembly CSIRO_AGI_GalMel_v1.^61^ BAM format files are worked on using samtools (v1.14).^60^ Bedtools^59^ (v2.30.0) was used for counting LQ-ssCAGE tags.

#### Computation and visualization of gene body coverage

The BAM files from LQ-ssCAGE and RNA-seq used as input to compute the gene body coverage yields by each library. SkewC^62^^,^^68^ workflow is used for the computation and visualization of gene body coverage. SkewC requires the reference transcript model for the *G. mellonella* (CSIRO_AGI_GalMel_v1_GCF_026898425.1_Annotation), which was downloaded from UCSC Genome browser.

#### TSS and enhancer peaks calling

To identify the promoters and enhancers, we created Biostrings-based genome data packages (BSgenome data packages) using BSgenomeForge Bioconductor R package (data and code availability). BSgenome data packages were used by CAGEfightR to identify the annotation of TSSs and enhancers.^29^ Also, we created an organism annotation package (OrgDb) for *G. mellonella* (data and code availability). BSgenome was used for annotation of the CAGE-defined TSSs and gene-level expression.

CAGEfightR analysis workflow performed as illustrated in (Figure 2).

1 Importing CTSSs to CAGEfightR and quantify CTSS.

The mapped reads for each library were converted to CTSSs in BED format using an in-house script. The CTSS in BED format (file per sample) and Metadata text file (one row per sample) are imported by CAGEfightR. The imported CTSSs are converted to bigwig files using convertBED2BigWig() function. The imported CTSSs used to quantify the CAGE TSS across all samples (*n* = 72). the function quantifyCTSSs() quantify the CTSS and implemented as follows:

CTSSs <- quantifyCTSSs(plusStrand=bw_plus, minusStrand = bw_minus, design=MetaData, inputAssay = "counts", genome=genomeInfo).

CTSSs defined as “*the number of CAGE tag 5′-ends mapping to each basepair (bp) in the genome*”^29^

2 CTSS-level analysis to analyze the number of CAGE tags per CTSS

The raw CTSS object is further processed to calculate pooled CTSS. For each CTSS, counts were summarized and normalized by TPM. The normalized CTSS counts were used to calculate the pooled CTSS, or CTSS signal across all samples. To reduce noise, pooled CTSSs were further processed by filtering out single tags spread across the genome. The filtering was accomplished by removing CTSSs detected in only a single sample. The final set of pooled CTSSs were used for the analysis at the level of clusters of CTSSs (Promoters and enhancers identification) and analysis at the level of annotated genes.

CTSSs <- calcTotalTags(CTSSs, inputAssay="counts",outputColumn="totalTags")

CTSSs <- calcTPM(CTSSs, inputAssay="counts", outputAssay="TPM", outputColumn="subsetTags")

CTSSs <- calcTPM(CTSSs, inputAssay="counts", outputAssay="TPM", totalTags="totalTags" )

CTSSs <- calcPooled(CTSSs, inputAssay="TPM")

CTSSs <- calcSupport(CTSSs, inputAssay="counts", outputColumn="support", unexpressed=0)

The above scripts resulted in total of 1,219,124 CAGE Transcription start sites in all 72 samples. The majority of CTSSs are only expressed in a single sample. Therefore, we discard these sites using subset, and then recalculate TPM values:

CTSSs <- subsetBySupport(CTSSs, inputAssay="counts", unexpressed=0, minSamples=2)

CTSSs <- calcTPM(CTSSs, totalTags="totalTags")

CTSSs <- calcPooled(CTSSs)

The sub-setting of the CTSS results in a final set of 332,601 across all samples.

3 Unidirectional and bidirectional clustering for finding TSS and enhancer candidates

Cluster-level analysis takes the results of CTSS-level analysis to identify clusters of CTSSs. It identifies two types of clusters, uni-directional (candidate TSSs) and bi-directional (candidate enhancers). We first identified the TSS

TCs <- quickTSSs(CTSSs)

TSSs <- TCs %>% calcTPM() %>% subsetBySupport(inputAssay="TPM", unexpressed=1, minSamples=4)

The above scripts resulted in results in total of 40,200 tag clusters (TCs) and 27,942 permissive TSS peaks. As explained in the result section, the permissive TSS peaks are filtered and only keep peaks that are expressed in all sample. The filtering of TSS peaks results in a set of robust TSS peaks.

Next, we identified the bidirectional clusters (candidate enhancers).

BCs <- quickEnhancers(CTSSs)

BCs <- subsetBySupport(BCs, inputAssay="counts", unexpressed=0, minSamples=4)

We obtained total of 728 enhancer peaks, which was filtered (only kept the intronic and exonic) to obtained 141.

#### Annotation of CAGE TSS, tag clusters, TSS and enhancer peaks

RefSeq annotation summary for *G. mellonella* genome assembly CSIRO_AGI_GalMel_v1 gen in (Figure S3). The GTF file downloaded from NCBI and used to create TxDB object (GenomicFeature).

txdb <- makeTxDbFromGFF(file = "AGI_GalMel_v2/data/GCF_026898425.1_CSIRO_AGI_GalMel_v1_genomic.gtf",organism = "Galleria mellonella",format = "gtf").

The txdb object was used to obtain the five genomic features (5′ UTR, CDS, intron, intergenic, and 3′ UTR).

   promoters_gr <- promoters(genes(txdb), upstream = 1000, downstream = 100)

          exons_gr <- exons(txdb)

       introns_gr <- unlist(intronsByTranscript(txdb))

      utr3_gr <- unlist(threeUTRsByTranscript(txdb))

       utr5_gr <- unlist(fiveUTRsByTranscript(txdb))

     cds_gr <- cdsBy(txdb, by = “gene”, use.names = FALSE) |> unlist

A summary of the five genomics features in (Figure S5).

To confirm the defining genomic features captured by CAGE (LQ-ssCAGE), CAGE TSS (CTSS), tag clusters (TC), TSS peaks, and enhancer clusters were intersected with the *G. mellonella* gene model for unified genomic annotation and characterization. Two functions from CageFightR used for the intersection of CTSS, TC, TSS and enhancers peaks with the genome model; assignTxID() annotate the peak with transcript IDs and assignTxType() annotate the peak with transcript context. To confirm the dinucleotide sequence of the dominant CTSS in, the dinucleotide frequencies spanning the −1/+1 positions relative to each CTSS peaks we extracted from the *G. mellonella* genome assembly fasta file (GCA_026898425.1_CSIRO_AGI_GalMel_v1_genomic.fna). For each of the features in (Figure 3D), the dinucleotide frequencies were calculated separately and presented as percentage withing each genomic feature.

#### Analysis of GC content bias for TSS peaks

To investigate the GC content bias in the identified TSS peaks (Figure 4D), we used the function addGCBias() from the R Bioconductor package chromVAR. The same Fasta file from the *G. mellonella* genome assembly (GalMel V1) were used. addGCBias () estimate the sequence GC content. The distribution of GC biased illustrated in log10 scale and divided by transcript type annotation associated with TSS peaks.

#### Creating genomic views of TSS and enhancer peaks

CageFightR R provide several functions to create genomic views of TSS and enhancer peaks. CageFightR uses Gviz R Bioconductor package for plotting genome-browser plot of TSSs and enhancers. The process includes creation of several tracks as documented in CageFightR. These tracks include, Genome track, Annotation track (using the txdb object created from the gtf file), CTSS track, and cluster track. After identifying the plot region, the plotTracks() used to combine them. plotTracks (list(axis_track, ctss_track, cluster_track, tx_track).

#### Identification of broad and sharp promoter and alternative usage of TSS

A common analysis of the data generated by any version of CAGE protocol is to investigate the presence of two types of TSS promoters, the Sharp and Broad classes. The division of TSS promoters by shape (broad and sharp) facilitated by CAGEfightR, it calculates promoter shape statistics which reveal information about the shape of identified promoter. The main strategy uses the Interquartile Range (IQR) to identify sharp and broad promoters. As recommended by CAGEfightR, we selected highly expressed TSSs (average TPM ≥ 10) which resulted in (Figure 5A).

We further characterized the main features of the identified broad and sharp promoter (TATA-box and CpG island), By plotting the sequence logo 50bp around the peaks (Figure 5B). In (Figure 5C) we plot the density distribution of the percentage of GC content and the position of the relative TSS. We tested for differential TSS usage (i.e., genes with multiple TSSs) as supported by CAGEfightR (Figure 5F).

#### Identification of interacting TSS and enhancers in G. mellonella

As reported in CAGE data, TSSs and enhancers that are in close proximity to each other and highly correlated (in expression) are candidates for physical interactions in CAGE data. CAGEfightR uses the genomics distance and expression correlation between identified TSSs and enhancers and identify TSSs-enhancer links as candidates. We utilized this function in CAGEfightR and identified it as set of TSS and enhancers interaction (Figure 6A). We identified the difference in expression between interacting and non-interaction TSS by plotting their expression in TPM (Figure 5B).

#### Prediction of the TFBS

To predict transcription factor binding sites for *G. Mellonella*, we used findMotifsGenome.pl from HOMER.^47^ The input for the command is the top 5% expressed TSS peaks. Also, the Homer command requires a FAST and GFF file for the *G. mellonella* genome, which were obtained from Genome assembly CSIRO_AGI_GalMel_v1 available from NCBI Genome.

To reduce the possibility of false positive call of the TF, we first extracted the top 5% highly expressed TSS peaks (1,866:37,320). The selected peaks were stored in a 6 columns BED file. Using awk linux command we converted the BED file into 2 bp TSS centered intervals. The processed BED, the genomic fasta file (GCF_026898425.1_CSIRO_AGI_GalMel_v1_genomic.fna) are used as input for the findMotifsGenome.pl command. The raw output of HOMER command is the Known motif and the HOMER *de novo* motifs (data and code availability). In our analysis we focused on the *de novo* HOMER motifs (Figure 7A). For the predicted TF we obtained their orthologs using NCBI Blast and analyzed their enrichment (Figure 7B) and their CAGE gene expression (Figure 7C).

#### Creating genomic views of the G. mellonella new annotation

To create ZENBU genome browser views, first, we fetched the FASTA format DNA and protein sequence alignment file (fna) for the Genome assembly CSIRO_AGI_GalMel_v1. The.fna file was downloaded from: https://ftp.ncbi.nlm.nih.gov/genomes/all/GCF/026/898/425/GCF_026898425.1_CSIRO_AGI_GalMel_v1/GCF_026898425.1_CSIRO_AGI_GalMel_v1_genomic.fna.gz.

Next, we retrieved the annotation files (.gff) for the assembly ASM364042v2 from: https://ftp.ncbi.nlm.nih.gov/genomes/all/GCF/026/898/425/GCF_026898425.1_CSIRO_AGI_GalMel_v1/GCF_026898425.1_CSIRO_AGI_GalMel_v1_genomic.gtf.gz.

The uploaded files were checked for completeness by searching randomly for several genes and confirming their annotation. Next, we uploaded the 72 aligned LQ-ssCAGE tag counts (stored in BAM format). Each BAM represents a single sample.

Sample metadata used for grouping of samples in ZENBU and other plots provided in (data and code availability).

### Quantification and statistical analysis

We used R statistics package for the processing, analysis, and plotting the figures.
