# Supplementary material for: Genome-wide mapping of the Galleria mellonella transcription start sites during fungal infection and treatment
Source: iScience. 2026 Oct 1;29(10):117675. doi: 10.1016/j.isci.2026.117675 (PMC13657207; doi:10.1016/j.isci.2026.117675)
Supplement: Supplementary file 1 [file mmc1.pdf]

**iScience, Volume *29***

## Supplemental information

# Genome-wide mapping of the *Galleria mellonella* transcription start sites during fungal infection and treatment

**Imad Abugessaisa, Mickey Konings, Ri-ichiroh Manabe, Michihira Tagami, Jessica Severin, Akira Hasegawa, Tsugumi Kawashima, Hiroko Kinoshita, Shohei Noma, Chitose Takahashi, Annelies Verbon, Yasushi Okazaki, Wendy W.J. van de Sande, and Takeya Kasukawa**

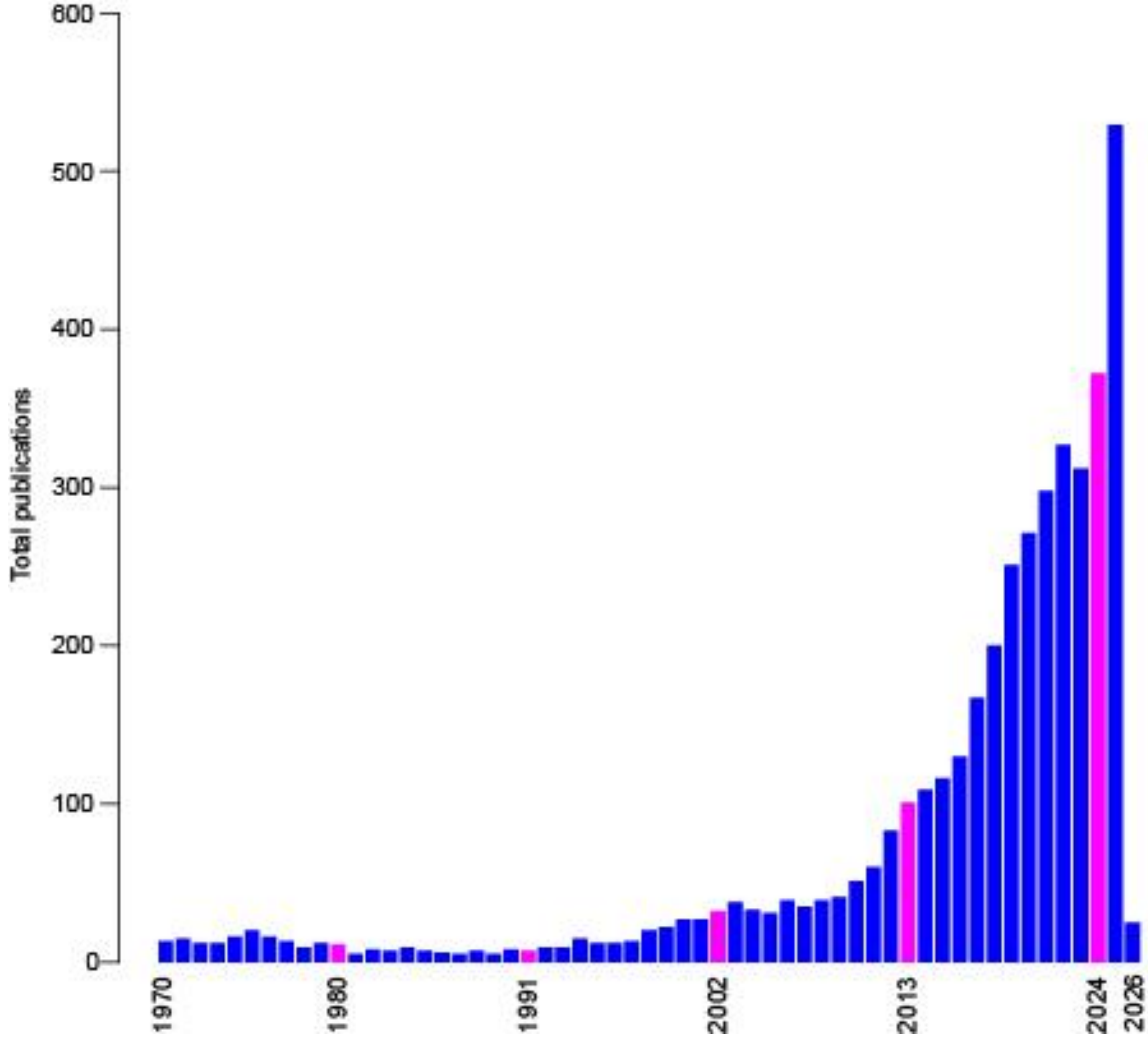


**Figure S1. Number of *Galleria mellonella*-related PubMed articles published between 1970 and 2026.** The figure highlights the growing use of *G. mellonella* as a disease model in biological research (PubMed search conducted on 22 January 2026).

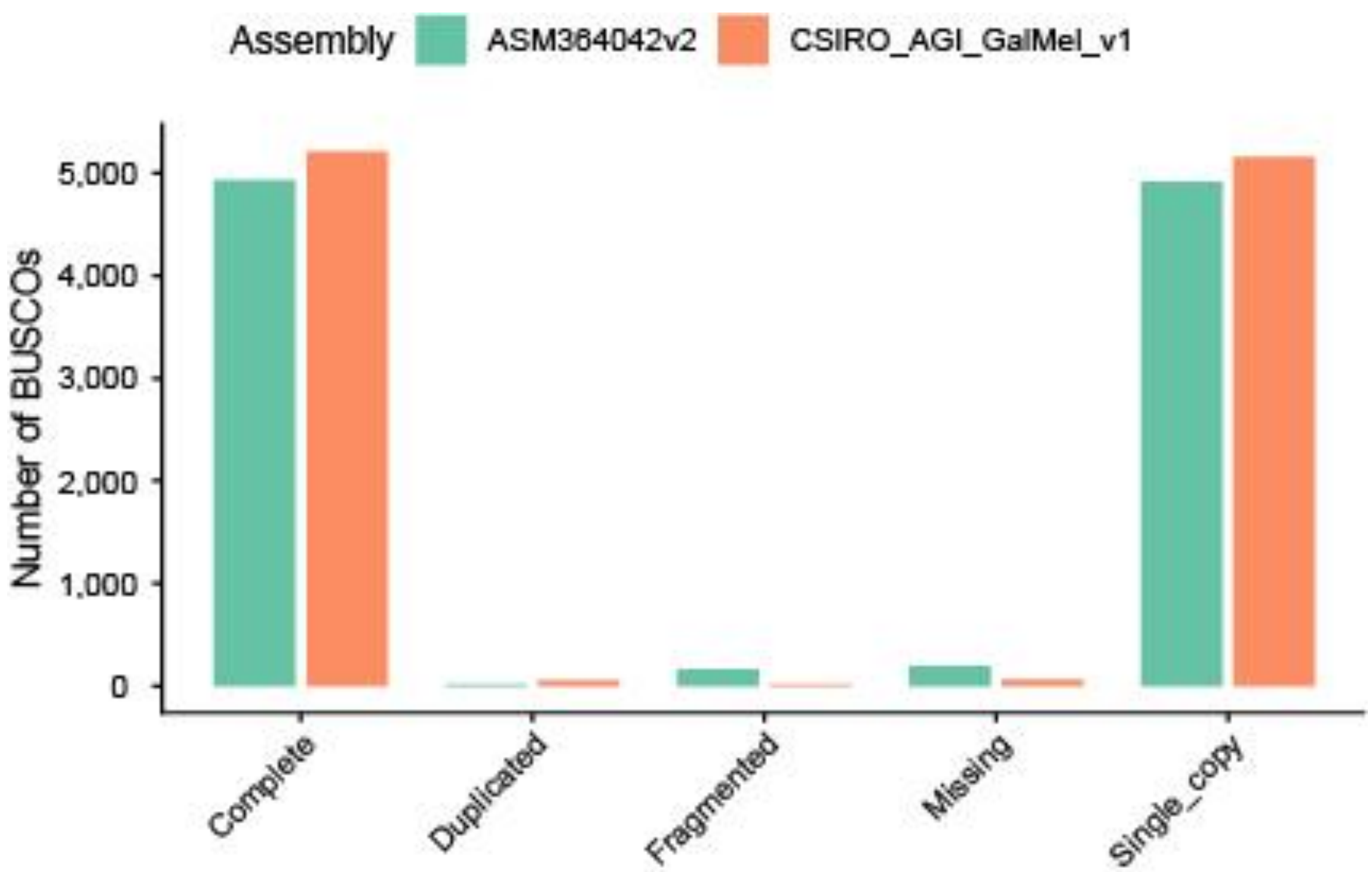


**Figure S2. BUSCO summary comparison of the two *Galleria mellonella* genome assemblies.** The figure highlights improvements in assembly completeness and genome coverage.

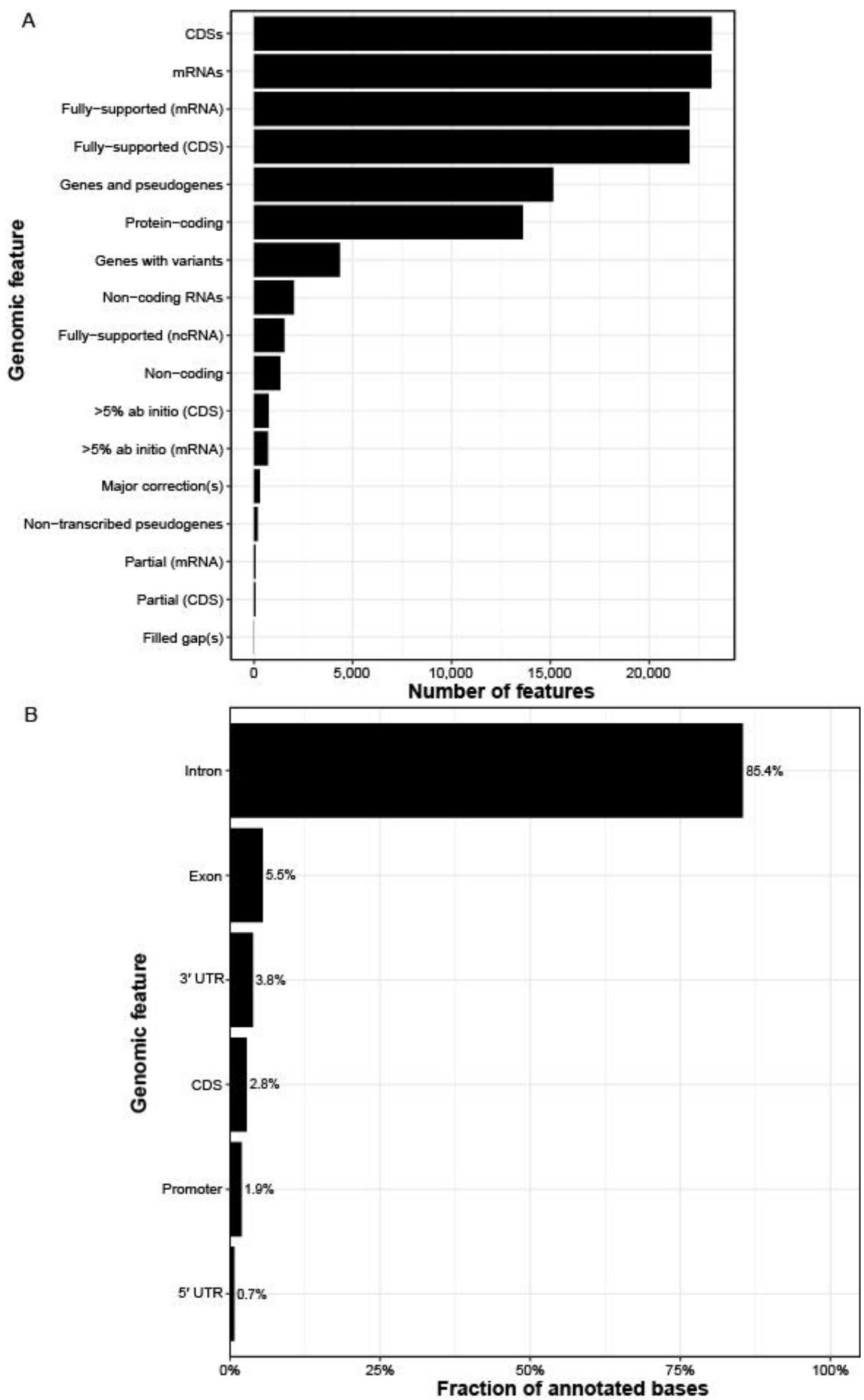


**Figure S3. Current gene model and biotypes annotation in *G. mellonella* CSIRO_AGI_GalMel_v1 genome assembly**. A. Biotypes annotation B. fraction on annotated bases under different genomic features

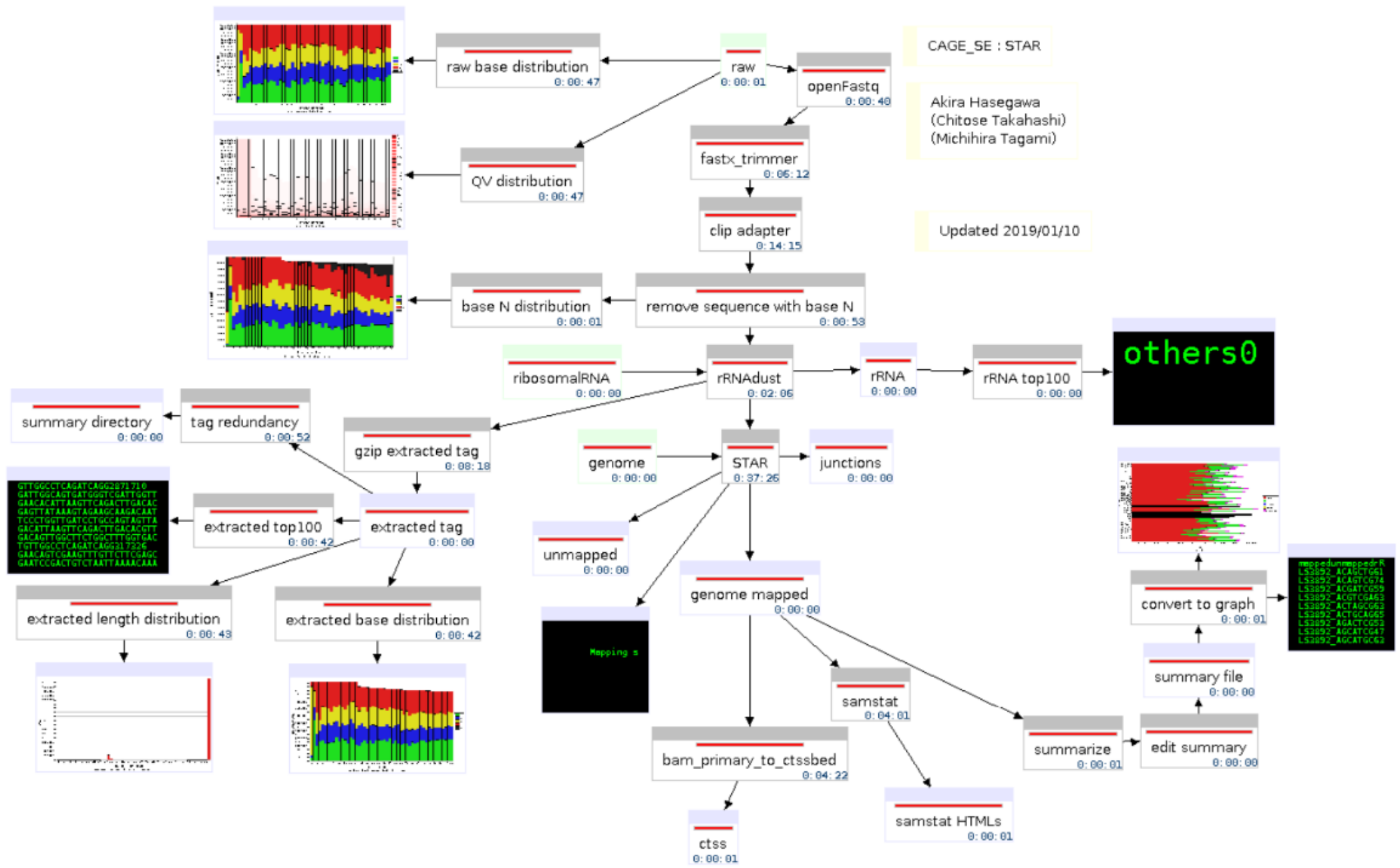


**Figure S4. LQ-ssCAGE data processing**. LQ-ssCAGE Paired-End sequence data provide cDNA sequence as Read1 and Sample Barcode sequence as Read2. In order to demultiplex Sample Barcode sequence, house made script is used with Read1 and Read2 data. Analysis of LQ-ssCAGE tags is performed by Moirai workflow using Read1 cDNA read.

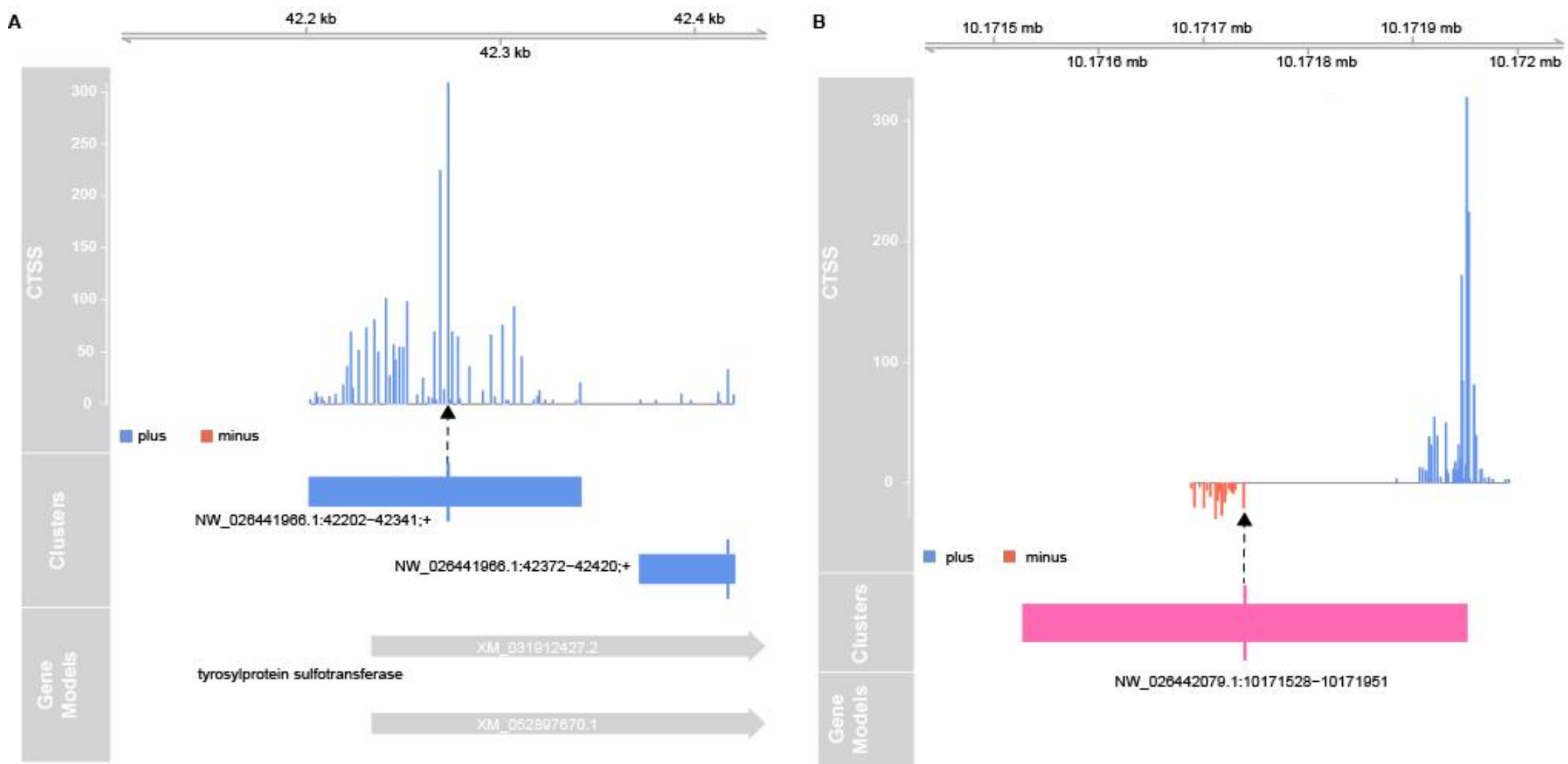


**Figure S5. Genome browser view.** Genome browser view of TSS peaks and enhancers. The genome browser view consists of three tracks. The top track shows the pooled CTSS signal and the middle track (clusters) shows TSS clusters with different annotations. Promoter peak type NW_026441966.1:42202-42341;+ and fiveUTR peak type NW_026441966.1:42372-42420;+. The gene model track at the bottom of the view shows the tyrosylprotein sulfotransferase gene with two transcripts. The vertical thick bar in the cluster indicates the most used CTSSs within the NW_026441966.1:42202-42341;+ cluster. The dashed arrows connect the tick bar to the TSS peak with the most used CTSS. Additional genomic views of TSS provided as (**Data S15**). H. Genome browser view of bidirectional cluster (enhancer NW_026442079.1:10171528−10171951). The vertical thick line in the cluster indicates the maximally balanced point within the cluster. The view shows the main characteristic of the active enhancer (the bidirectional pattern of the expression in both the plus and minus strands). Additional view provided as (**Data S16**).

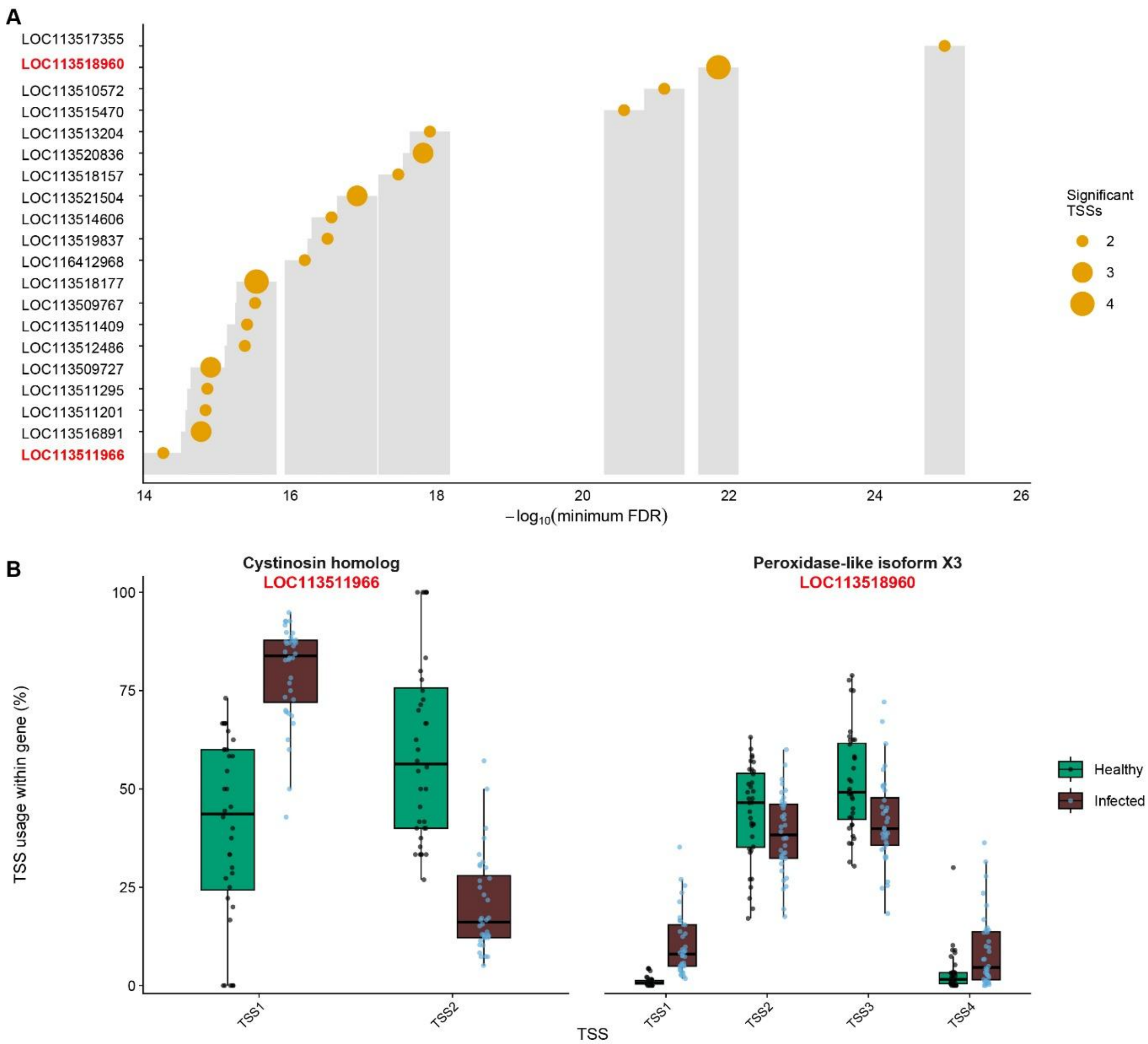


**Figure S6. Differential TSS usage analysis.** Figure S6. Differential TSS usage between Healthy and Infected samples. Box plots are colored by the treatment condition. Points in the box plots represent library.

**A**. Top-ranked Galleria mellonella genes showing evidence of differential TSS usage between Healthy and Infected samples. Genes are ranked by −log10 minimum FDR, and point size indicates the number of significant TSSs detected for each gene. Highlighted genes, LOC113511966 (Cystinosin homolog) and LOC113518960 (Peroxidase-like isoform X3), were selected for detailed visualization.

**B**. Relative usage of alternative TSSs within two representative genes, LOC113511966 (Cystinosin homolog) and LOC113518960 (Peroxidase-like isoform X3), in Healthy and Infected samples. Boxplots show the percentage contribution of each TSS to total gene-level TSS usage, with individual samples overlaid as points. The observed shifts indicate condition-associated changes in promoter/TSS selection during infection.

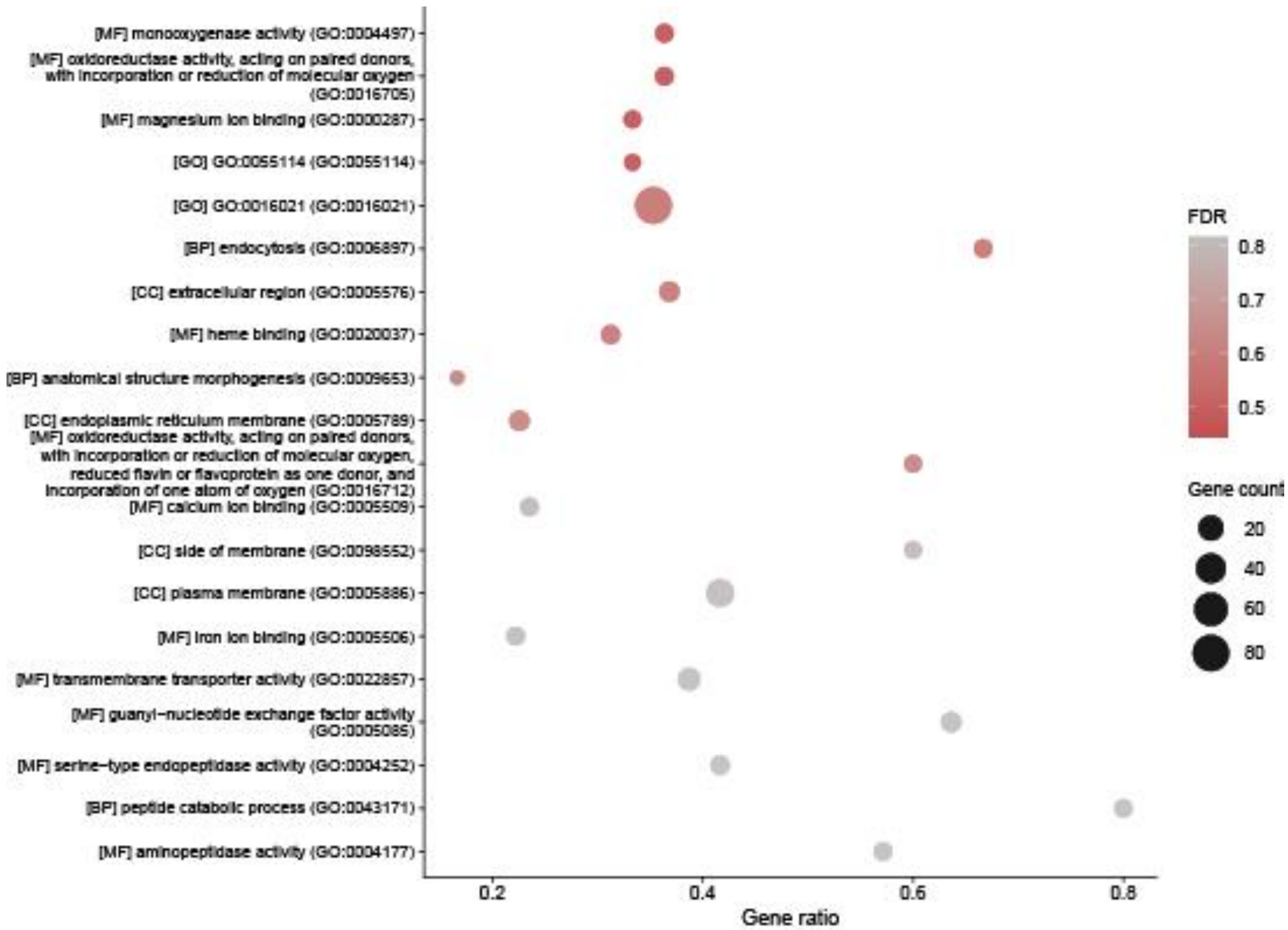


**Figure S7. GO enrichment analysis of genes associated with differential TSS usage.** Pre-ranked GO enrichment analysis was performed using genes associated with differential TSS usage between Healthy and Infected samples. The x-axis shows the gene ratio, and point size indicates the number of genes associated with each GO term. Point color represents the FDR value. Although several GO terms related to membrane processes, transporter activity, ion binding, enzymatic activity, and metabolic functions were detected among the top-ranked terms, none passed the significance threshold of FDR < 0.05. Therefore, these results should be interpreted as exploratory.

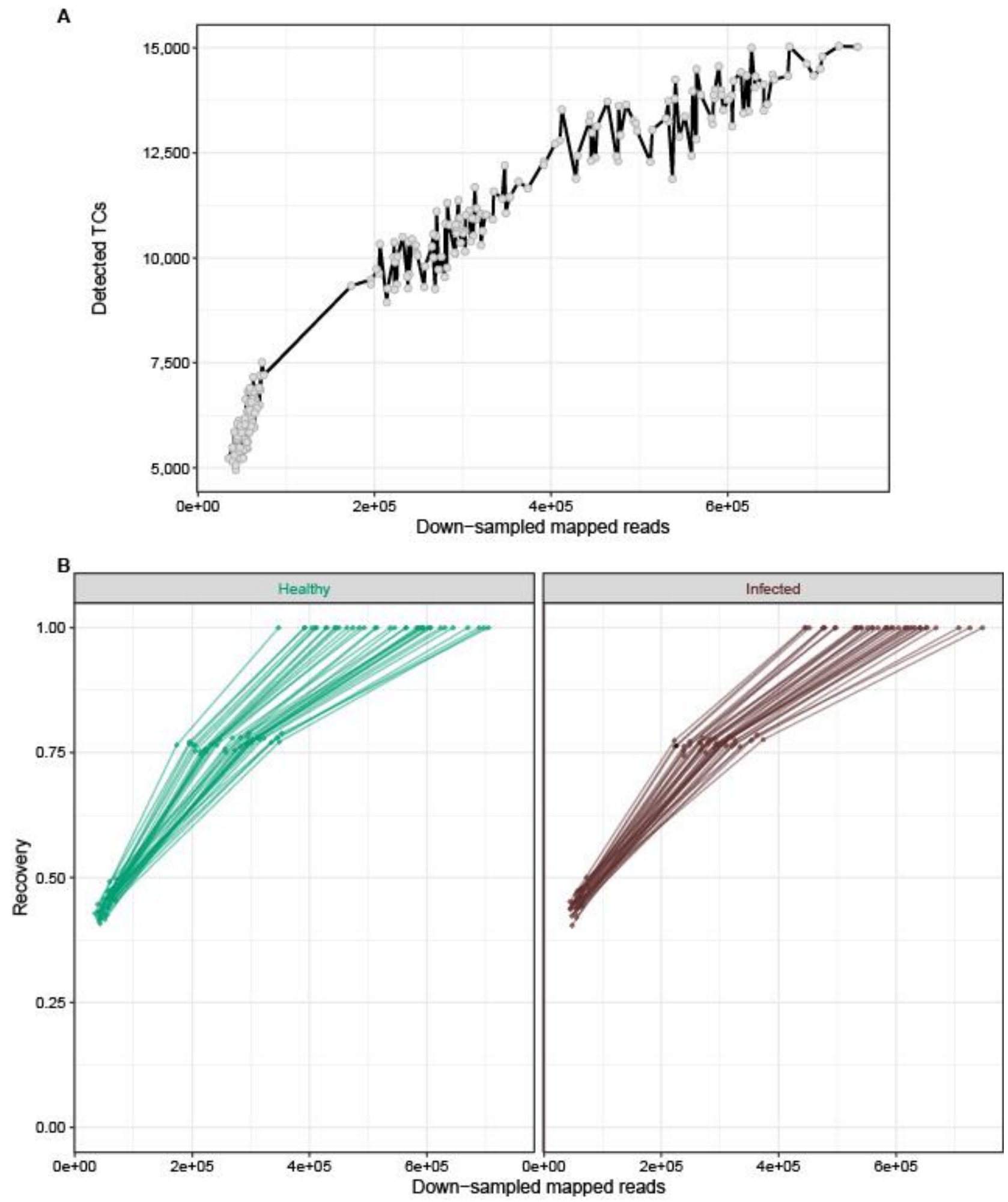


**Figure S8. Saturation analysis.** Impact of sequence depth on the detected tag clusters (TC). A. Sample−level TC. X-axis: down-sampled mapped reads (effective read depth after keeping a fraction of reads). Y-axis: number of detected TCs at that depth. B. Sample−level TC recovery by Condition. Each thin line is one sample / library and shows how many of that sample's full-depth TCs are recovered as we increase read depth by down-sampling. X-axis the number of mapped reads after down-sampling. Y-axis shows recovery as range 0–1 (TCs detected in down-sample and detected at full depth). In the plot 1.0 indicates the recovery of all TCs that were detectable in that sample at full depth.

**TableS1**: Basic QC information of the total RAN used to prepare LQ-ssCAGE libraries

| Sample No. | Sample name | Concentration (ng/ul) | Volume (ul) | Total amount of the sample (ug) | A260/280 | A260/230 |
|---|---|---|---|---|---|---|
| 1 | Control Healthy 1 6h | 138.4 | 40 | 5.5 | 2.11 | 1.36 |
| 2 | Control Healthy 2 6h | 466.7 | 40 | 18.7 | 2.15 | 1.99 |
| 3 | Control Healthy 3 6h | 195.4 | 40 | 7.8 | 2.10 | 2.41 |
| 4 | Control Infected 1 6h | 308 | 40 | 12.3 | 2.12 | 1.70 |
| 5 | Control Infected 2 6h | 390.2 | 40 | 15.6 | 2.13 | 1.75 |
| 6 | Control Infected 3 6h | 326.8 | 40 | 13.1 | 2.14 | 1.42 |
| 7 | Ravuconazole infected 1 6h | 562 | 40 | 22.5 | 2.17 | 2.10 |
| 8 | Ravuconazole infected 2 6h | 485.7 | 40 | 19.4 | 2.16 | 1.99 |
| 9 | Ravuconazole infected 3 6h | 372.3 | 40 | 14.9 | 2.15 | 1.63 |
| 10 | Ravuconazole control 1 6h | 321 | 40 | 12.8 | 2.13 | 1.37 |
| 11 | Ravuconazole control 2 6h | 482.6 | 40 | 19.3 | 2.15 | 1.96 |
| 12 | Ravuconazole control 3 6h | 241 | 40 | 9.6 | 2.13 | 2.38 |
| 13 | Itraconazole infected 1 6h | 588.3 | 40 | 23.5 | 2.13 | 2.38 |
| 14 | Itraconazole infected 2 6h | 352.3 | 40 | 14.1 | 2.14 | 2.35 |
| 15 | Itraconazole infected 3 6h | 520.5 | 40 | 20.8 | 2.15 | 2.03 |
| 16 | Itraconazole control 1 6h | 275 | 40 | 11.0 | 2.12 | 1.23 |
| 17 | Itraconazole control 2 6h | 70 | 40 | 2.8 | 2.07 | 1.06 |
| 18 | Itraconazole control 3 6h | 186.3 | 40 | 7.5 | 2.12 | 0.73 |
| 19 | Control Healthy 1 30h | 261.2 | 40 | 10.4 | 2.12 | 1.84 |
| 20 | Control Healthy 2 30h | 342.2 | 40 | 13.7 | 2.14 | 1.37 |
| 21 | Control Healthy 3 30h | 358.3 | 40 | 14.3 | 2.15 | 1.61 |
| 22 | Control Infected 1 30h | 487.5 | 40 | 19.5 | 2.18 | 2.27 |
| 23 | Control Infected 2 30h | 519 | 40 | 20.8 | 2.17 | 2.27 |
| 24 | Control Infected 3 30h | 366.1 | 40 | 14.6 | 2.12 | 1.18 |
| 25 | Ravuconazole infected 1 30h | 406.5 | 40 | 16.3 | 2.12 | 1.86 |
| 26 | Ravuconazole infected 2 30h | 291.2 | 40 | 11.6 | 2.10 | 1.80 |
| 27 | Ravuconazole infected 3 30h | 503.3 | 40 | 20.1 | 2.18 | 1.69 |
| 28 | Ravuconazole control 1 30h | 205.8 | 40 | 8.2 | 2.10 | 1.34 |
| 29 | Ravuconazole control 2 30h | 264.2 | 40 | 10.6 | 2.10 | 1.68 |
| 30 | Ravuconazole control 3 30h | 181.7 | 40 | 7.3 | 2.12 | 1.12 |
| 31 | Itraconazole infected 1 30h | 265.1 | 40 | 10.6 | 2.11 | 1.72 |
| 32 | Itraconazole infected 2 30h | 248.1 | 40 | 9.9 | 2.13 | 1.65 |
| 33 | Itraconazole infected 3 30h | 341.9 | 40 | 13.7 | 2.14 | 2.26 |
| 34 | Itraconazole control 1 30h | 113.5 | 40 | 4.5 | 2.13 | 0.23 |
| 35 | Itraconazole control 2 30h | 288 | 40 | 11.5 | 2.13 | 2.07 |
| 36 | Itraconazole control 3 30h | 178.7 | 40 | 7.1 | 2.10 | 1.66 |
| 37 | Control Healthy 1 72h | 303.5 | 40 | 12.1 | 2.13 | 0.69 |

| | | | | | | |
|---|---|---|---|---|---|---|
| 38 | Control Healthy 2 72h | 258.7 | 40 | 10.3 | 2.12 | 1.50 |
| 39 | Control Healthy 3 72h | 410.1 | 40 | 16.4 | 2.13 | 2.10 |
| 40 | Control Infected 1 72h | 364.6 | 40 | 14.6 | 2.13 | 1.67 |
| 41 | Control Infected 2 72h | 495.2 | 40 | 19.8 | 2.16 | 2.40 |
| 42 | Control Infected 3 72h | 470.3 | 40 | 18.8 | 2.13 | 2.13 |
| 43 | Ravuconazole infected 1 72h | 174.3 | 40 | 7.0 | 2.11 | 0.83 |
| 44 | Ravuconazole infected 2 72h | 359.2 | 40 | 14.4 | 2.11 | 1.97 |
| 45 | Ravuconazole infected 3 72h | 221.6 | 40 | 8.9 | 2.09 | 1.74 |
| 46 | Ravuconazole control 1 72h | 77.7 | 40 | 3.1 | 2.05 | 0.67 |
| 47 | Ravuconazole control 2 72h | 222.3 | 40 | 8.9 | 2.12 | 0.73 |
| 48 | Ravuconazole control 3 72h | 328 | 40 | 13.1 | 2.13 | 2.05 |
| 49 | Itraconazole infected 1 72h | 472.3 | 40 | 18.9 | 2.14 | 2.38 |
| 50 | Itraconazole infected 2 72h | 552 | 40 | 22.1 | 2.14 | 1.63 |
| 51 | Itraconazole infected 3 72h | 384.1 | 40 | 15.4 | 2.14 | 2.18 |
| 52 | Itraconazole control 1 72h | 234.1 | 40 | 9.4 | 2.11 | 1.91 |
| 53 | Itraconazole control 2 72h | 105.1 | 40 | 4.2 | 2.08 | 0.59 |
| 54 | Itraconazole control 3 72h | 368 | 40 | 14.7 | 2.13 | 1.97 |
| 55 | Control Healthy 1 168h | 264.2 | 40 | 10.6 | 2.14 | 1.38 |
| 56 | Control Healthy 2 168h | 371.5 | 40 | 14.9 | 2.14 | 1.85 |
| 57 | Control Healthy 3 168h | 440.9 | 40 | 17.6 | 2.16 | 2.13 |
| 58 | Control Infected 1 168h | 469.1 | 40 | 18.8 | 2.14 | 1.86 |
| 59 | Control Infected 2 168h | 332.5 | 40 | 13.3 | 2.14 | 2.27 |
| 60 | Control Infected 3 168h | 503.1 | 40 | 20.1 | 2.11 | 2.50 |
| 61 | Ravuconazole infected 1 168h | 84.2 | 40 | 3.4 | 2.09 | 0.35 |
| 62 | Ravuconazole infected 2 168h | 167 | 40 | 6.7 | 2.11 | 1.52 |
| 63 | Ravuconazole infected 3 168h | 236.1 | 40 | 9.4 | 2.10 | 1.96 |
| 64 | Ravuconazole control 1 168h | 513.8 | 40 | 20.6 | 2.14 | 1.69 |
| 65 | Ravuconazole control 2 168h | 580.9 | 40 | 23.2 | 2.15 | 1.92 |
| 66 | Ravuconazole control 3 168h | 504.5 | 40 | 20.2 | 2.14 | 1.89 |
| 67 | Itraconazole infected 1 168h | 204.6 | 40 | 8.2 | 2.10 | 0.76 |
| 68 | Itraconazole infected 2 168h | 207.2 | 40 | 8.3 | 2.11 | 1.09 |
| 69 | Itraconazole infected 3 168h | 354.5 | 40 | 14.2 | 2.14 | 0.76 |
| 70 | Itraconazole control 1 168h | 641.2 | 40 | 25.6 | 2.15 | 1.80 |
| 71 | Itraconazole control 2 168h | 467.6 | 40 | 18.7 | 2.16 | 1.47 |
| 72 | Itraconazole control 3 168h | 756.4 | 40 | 30.3 | 2.13 | 2.37 |

**TableS2**: Description of the supplementary data provided in Mendeley data

| Category | File | Description |
|---|---|---|
| BAM | GM_TSS_Atlas_TSS_BAMs.tar.gz | Data S3 |
| Gene Body Coverage | RNA-seq_Gene_Body_Coverage.r.zip | Data S1 |
| Gene Body Coverage | LQ-ssCAGE_Gene_Body_Coverage.r.zip | Data S2 |
| CTSS | GM_TSS_Atlas_Raw_CTSSs.v2.rds.gz | Data S4 |
| CTSS | GM_TSS_Atlas_Filtered_CTSSs.v2.rds.gz | Data S5 |
| Tag clusters | GM_TSS_Atlas_TCs_Robject_v2.rds.gz | Data S6 |
| TSS | GM_TSS_Atlas_Permissive_TSS_Peaks_Robject_v2.rds.gz | Data S7 |
| TSS | GM_TSS_Atlas_Robust_TSS_Peaks_Robject_v2.rds.gz | Data S8 |
| Enhancers | GM_TSS_Atlas_Raw_Enhancers_Robject_v2.rds.gz | Data S9 |
| Enhancers | GM_TSS_Atlas_Filtered_Enhancers_Robject_v2.rds.gz | Data S10 |
| GTF | GCF_026898425.1_CSIRO_AGI_GalMel_v1_GM_TSS_Atlas_TSS_Enhancers.v2.Permissive.gtf.gz | Data S11 |
| GTF | GCF_026898425.1_CSIRO_AGI_GalMel_v1_GM_TSS_Atlas_TSS_Enhancers.v2.Robust.gtf.gz | Data S12 |
| GTF | GM_TSS_Atlas_TSS_Enhancers.v2.Permissive.gtf.gz | Data S13 |
| GTF | GM_TSS_Atlas_TSS_Enhancers.v2.Robust.gtf.gz | Data S14 |
| HOMER Motif Analysis | Motif_Analysis_Files.tar.gz | Data S15 |
| HOMER Motif Analysis | GM_TSS_Atlas_HOMER_Motif_Enrichment.v1.tar.gz | Data S16 |
| R Bioconductor package | BSgenome.Gmellonella.NCBI.CSIRO.AGI.GalMel.v1.zip | Data S17 |
| R Bioconductor package | org.GGalleriamellonella.eg.db.zip | Data S18 |
| Metadata | MetaData.zip | Data S19 |
| TSS | GM_TSS_Atlas_TSS_Genome_View.zip | Data S15 |
| Enhacners | GM_TSS_Atlas_Enhancers_Genome_View.zip | Data S16 |
